# Toward Sustainable Diets—Interventions and Perceptions Among Adolescents: A Scoping Review

**DOI:** 10.1093/nutrit/nuae052

**Published:** 2024-05-29

**Authors:** Adeline R Lanham, Jolieke C van der Pols

**Affiliations:** School of Exercise and Nutrition Sciences, Faculty of Health, Queensland University of Technology, Brisbane, Queensland, 4059, Australia; Centre for Agriculture and the Bioeconomy, Queensland University of Technology, Brisbane, Queensland, 4000, Australia; School of Exercise and Nutrition Sciences, Faculty of Health, Queensland University of Technology, Brisbane, Queensland, 4059, Australia; Centre for Agriculture and the Bioeconomy, Queensland University of Technology, Brisbane, Queensland, 4000, Australia

**Keywords:** sustainable diets, adolescents, nutrition interventions, perceptions of diets

## Abstract

Adolescence is an important life stage during which shifts toward more healthy and sustainable diets can be promoted. Adolescents have increasing influence over their food choices, informed by their developing personal knowledge and values, affecting long-term dietary behaviors into adulthood. The recent literature regarding adolescents’ (1) perceptions of environmentally sustainable diets and (2) interventions to support adolescents to eat sustainably was reviewed in this study. We reviewed published literature that focused on adolescent participants and their perceptions of, or interventions to support, sustainable dietary habits. Five electronic databases were searched to include studies published since 2012 that met the inclusion criteria, including reporting of participants aged between 11 and 18 years, reporting on adolescents’ perceptions of sustainable diets or interventions implemented to improve the sustainability of adolescents’ diets, and framed in the context of sustainability. Data were extracted, including study and participant characteristics, methodology, and results in relation to each of the 2 research focus areas. Twenty-eight articles were included in the review. Findings suggest that adolescents’ understanding of what constitutes sustainable eating is poor. Adolescents who had previously received education regarding sustainable diets valued nature and health, or were from a rural or indigenous community, were more likely to value environmentally sustainable-food choices. Interventions which target adolescents’ understanding of and aspiration to make sustainable-food choices appears to improve their attitudes toward sustainable food, whereas interventions to increase the availability of sustainable foods improved the environmental sustainability of adolescents’ dietary intake. Multicomponent, tailored, and community-based interventions were most effective; however, the long-term effect of these interventions remains unclear. More research is needed in low- and middle-income countries, with consideration of adolescents’ level of autonomy in food choice in local food environments and the long-term effectiveness of interventions.

**Systematic Review Registration**: Open Science Framework identifier osf.io/h3jz6.

## INTRODUCTION

Shifts toward more sustainable diets and food systems are needed to reduce impacts on the natural environment. Sustainable diets are defined by the UN Food and Agriculture Organization (FAO) as “dietary patterns that promote all dimensions of individuals’ health and well-being; have low environmental pressure and impact; are accessible, affordable, safe and equitable; and are culturally acceptable.”^1^

An estimated 35% of total global greenhouse gas emissions (GHGE) is associated with food production and consumption,^2^ but it has been estimated that dietary change could reduce the environmental impact (land use and GHGE) of the food system by up to 50%.^3^ Food choices and dietary habits also need to become healthier, including those of young people, because adolescents have some of the poorest diets of all life stages,^4^ and 18% of children and adolescents globally (n = 340 million) are overweight or obese.^5^ To address these concerns, research is needed to inform and shift food systems to be more sustainable. Food systems are complex and dynamic, and consist of agricultural production, food transport and supply chains, food environments (eg, where people buy and consume foods), individual factors such as knowledge and values toward certain foods, and consumer behavior in relation to food purchasing and preparation.^6^^,^^7^ As outlined in the Food Systems Dashboard framework, such complex food systems have dietary and broader social, environmental, and economic outcomes.^6^ Because of their interactive nature, changes in certain processes of the food system have up- and downstream, and potentially compounding, effects. These invite the consideration of multiple strategies to improve the sustainability of food systems.

Food habits are influenced and formed during adolescence as young people engage with food environments with increasing autonomy (ie, independent decision making) and agency (ie, ability to carry out these decisions) as they grow older.^8^^,^^9^ During adolescence, food choices may be influenced by sociocultural context, the physical environment, immediate social relationships, and individual factors (eg, knowledge, skills, and attitudes).^10^ Each of these factors may be targeted by interventions to promote dietary improvements,^11^ including those that promote environmental sustainability. The high level of awareness among young people that climate change is an urgent global emergency makes environmentally sustainable-food choice a priority among the adolescent population.^12^

Personal perceptions, values, and attitudes develop during adolescence, such as a concern for personal health or environmental well-being. Food-related knowledge and skills may develop at school or in the home as adolescents take increasing responsibility for personal food choices.^13^ Previous research has suggested that major influences on adolescents’ consumption behaviors are driven by aspirational (ie, attitudes, values), cognitive (ie, knowledge), and situational (including social) factors.^13–15^ These factors may then influence dietary habits during adolescence or later into adulthood.^13^^,^^16^^,^^17^ The interaction among an adolescent’s individual characteristics, the food environment, and their food choices is important to understand when considering the health and sustainability of adolescents’ diets.

Adolescents are an important target group for efforts to improve sustainability of dietary habits. Some studies addressing sustainable dietary habits and values of adolescents have been published. To support future research and strategies in this field, we conducted a scoping review to summarize available evidence and identify current gaps. Therefore, in this scoping review, we aimed to summaries global evidence in 2 focus areas: (1) adolescents’ perceptions of sustainable diets and (2) interventions that have been implemented to improve the sustainability of adolescents’ diets.

## METHODS

The process of this scoping review was guided by the JBI methodology for scoping reviews^18^ and Preferred Reporting Items for Systematic Reviews and Meta-Analyses (PRISMA) checklist extension for scoping reviews.^19^ In brief, this involved the development of research objectives and inclusion criteria, and undertaking planned information searching, selection, extraction, analysis, and presentation, including implications.^18^^,^^19^ A registered protocol was lodged with Open Science Framework (OSF) in September 2022 (OSF identifier osf.io/h3jz6; https://osf.io/h3jz6/).

### Search strategy, screening, and selection of evidence

Preliminary searches were carried out in 3 electronic databases (PubMed, Web of Science, and Embase) to identify appropriate literature. Results from this preliminary search were used to further identify relevant search terms and define the scope of the review in consultation with a collaborating librarian. The full search strategy, including the 5 main concepts (adolescent, diet, environmental sustainability, intervention, and perceptions; see Table S1 for more details), was adapted for 6 electronic databases (PubMed, Scopus, Embase, Web of Science, ERIC, and Education Source). Searches were narrowed to literature published between January 2012 and December 2022, given the rapidly changing food supply and environments,^20^ and attitudes toward environmental sustainability, particularly among youth.^21^ Non–peer-reviewed literature was included, given the limited published work in the study area, with evidence published in academic journals that were listed in the screened databases likely to be informative despite limitations. Following the full search, citations were exported to EndNote 20.0.1 (Clarivate, Philadelphia, PA) and duplicates removed prior to using JBI SUMARI (https://sumari.jbi.global/) for screening.

Inclusion criteria were developed to define the 5 main concepts and source types (Table 1). Adolescence was defined as age between 11 and 18 years because this is the period typically during which young people attend high school and experience important social, cognitive, and physical changes of adolescence.^11^ A total of 200 article titles and abstracts (∼10% of the total) were screened for inclusion by both review authors for consistency, with 1 of these researchers (A.R.L, lead author) screening the remainder of articles independently. All full-text articles were screened in duplicate by both reviewers, and discrepancies of inclusion were settled through discussions between them. The reference lists of included articles were screened for additional sources.

**Table 1.** Criteria for inclusion of studies

Participants	Most participants are aged between 11 and 18 y, or At least one-third of participants are aged between 11 and 18 y and data for participants within this age group are reported separately from those that are older or younger, and Participants reflect the nutrition and medical needs of the general population (eg, not focused on those with eating disorders, allergies, or other health conditions), and Adolescent participants are considered as consumers within the study (eg, not a primary role as food producers)
Concept	Include an intervention or strategy implemented with reference to improving the sustainability of adolescents’ dietary habits, or Gather adolescents’ perceptions, understanding, attitudes, and/or knowledge of sustainable dietary habits
Context	The study must refer to the environmental sustainability of diets or are framed within the context of sustainability (per the United Nations Food and Agriculture Organization definition,^114^ rather than longevity). This includes aspects of food literacy, food environments, food choice, preparation, consumption, and waste, that impact on the environment.
Sources	All types of sources will be included from the specified online databases. Published between January 2012 and December 2022 Available in English

### Data extraction, analysis, and presentation of results

Data were extracted from the included sources depending on the aspect of the research aims addressed in the study. For studies of adolescents’ perceptions of sustainable diets, data extracted included: first author’s surname, year of publication, country, context, participant characteristics, study aims, perceptions of sustainable eating, theory or framework used, methods, key findings, and conclusions. Extraction of data on perceptions of sustainable eating was conceptualized from the individual factors reported in the Food Systems Dashboard, a framework with global scope and traction.^6^ These factors are classified as cognitive knowledge and understanding, aspirational value, or perceived barriers to and enablers of sustainable dietary behaviors (mapped across all domains of the food system).^6^

For intervention studies, the following data were extracted: first author’s surname, year of publication, country, context, participant characteristics, intervention type, principles of sustainable diets addressed according to the FAO’s principles of sustainable diets,^1^ study aims, intervention components or types with consideration of the food system framework,^6^ theory or framework used, methods, key findings, and conclusions.

Critical appraisal of included articles was not undertaken; however, limitations of the included articles and the review methodology were considered. Extracted data, including limitations of each source, were tabulated using 2 Excel spreadsheets (1 for each of the research questions; Microsoft, Redmond, WA) (Tables S2 and S3). Finally, the extracted data were reported, synthesized, and discussed in the context of the food system framework and broader research to inform the implications of this review for future research.

## RESULTS

**Figure 1.**
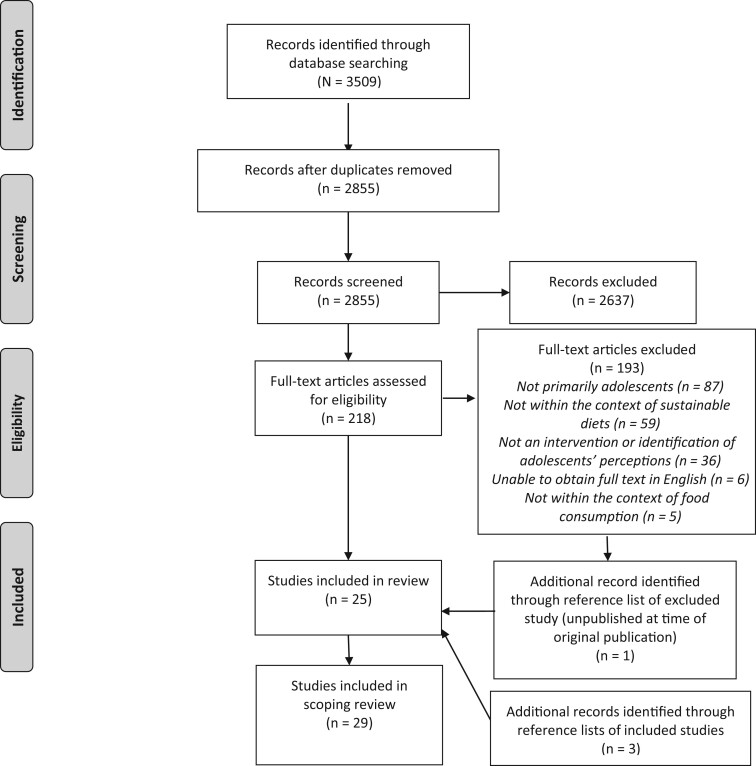
Preferred Reporting Items for Systematic Reviews and Meta-Analyses (PRISMA) flow diagram displaying screening and inclusion of studies

A total of 2843 articles’ titles and abstracts were screened, and 214 full-text articles were retrieved. Of these, 25 articles met the inclusion criteria. The primary reason studies were excluded was age of the study participants (n = 87 articles); however, articles were commonly excluded for more than 1 reason, with only the first identified reason reported. An additional 4 articles were included after screening of the titles of references from articles. In total, 2 articles were conference papers and the remaining 27 were journal articles. Figure 1 outlines the PRISMA flow diagram of this process.^22^

Nineteen of the included studies reported on adolescents’ perceptions, understanding, value, attitudes, or barriers to and enablers of consumption of sustainable foods. Sixteen of the included articles reported on intervention studies aimed at improving the sustainability of adolescents’ diets. A detailed summary of data extracted for each of these studies can be found in Tables S2 and S3. The findings from the review are discussed separately for each of the 2 focus areas.

### Adolescents’ perceptions of sustainable diets

#### Perception study characteristics

Of the 19 included studies reporting on adolescents’ perceptions of sustainable diets, 12 were cross-sectional studies (in which researchers reported on adolescents’ perceptions or intentions of sustainable eating from observation, without an intervention) and 7 were intervention studies (in which researchers implemented an intervention and assessed adolescents’ perceptions of sustainable diets before and/or afterward). A large number of articles (n = 8) were published after 2018. Studies were predominantly reports from Europe (n = 10; n = 5 from Sweden), with studies also from the Americas (n = 3; n = 1 from the Caribbean), Australia (n = 3), Asia (n = 2), New Caledonia (n = 1), and 1 study including participants from both the United Kingdom and Italy. Within and across these countries, the socioeconomic demographics of participants varied from those at risk of food insecurity in Alaska to high-income areas of China and Sweden (Table S2 for details). Most studies reported on adolescents’ understanding of sustainable diets (n = 13), perceived value, or importance of sustainable diets (n = 15), and perceived barriers and enablers to adhering to sustainable dietary behaviors (n = 11). We discuss each of these aspects of adolescents’ personal perceptions of sustainable diets.

#### Cognitive knowledge and understanding of sustainable diets

Adolescents’ understanding of sustainable diets generally appears to be narrow or lacking. Most adolescents, when asked, were unsure of what constitutes sustainable eating,^23^ or a plant-based diet.^24^ The complexity of sustainability has been reported as barrier to understanding sustainable eating.^25^ This notion could explain findings by Gisslevik et al,^26^ who reported that adolescents displayed limited understanding of the sustainability of foods from a systemic or complex approach (eg, food systems, complexity of environmental impact), with greater understanding of aspects more tangible to individuals, such as health impacts and cost.

However, the included studies indicate that many adolescents understand the principles of sustainable diets or can provide examples of sustainable dietary behavior. In relation to the FAO’s principles of a sustainable diet, when discussing sustainable aspects of foods, adolescents most commonly mentioned those related to health rather than the environment.^27^ Other aspects of sociocultural and economic sustainability were mentioned even less frequently and almost always after mention of either health and or economic aspects.^28^ When describing or defining environmental sustainability of foods, some adolescents described it as good for the environment, planet, and/or climate.^23^ Some adolescents reported misconceptions, including sustainable food not being connected to environmental sustainability, and others reported that ecological sustainability is incompatible with economic sustainability.^27^

When determining a food’s sustainability, adolescents often referred to production and/or transport characteristics, such as the location or proximity of production,^23^^,^^28–33^ organic nature of production,^26^^,^^29–31^^,^^34^ or animal welfare considerations (eg, cage-free eggs, free-range meat).^30^^,^^33^ A few studies reported that the types of foods consumed, such as a reduced intake of animal-sourced foods, contributed to the environmental sustainability of a diet and often had health benefits.^26^^,^^35^ Some adolescents also mentioned the consideration of packaging types and reducing food waste as aspects of sustainable diets.^23^^,^^35^ When identifying if a particular product, including food, was sustainable, participants looked to the packaging for information.^23^^,^^30^ The environmental impact of the production methods, transport, and packaging of foods was most commonly reported when adolescents considered the environmental impact of their foods.

#### Aspirational value of sustainable diets

When reporting on factors that influence food choice, most studies reported that sustainability was not a strong consideration.^23^^,^^25^ Taste was a dominant factor,^35^ as well as the food’s healthiness.^23^^,^^36^ This was demonstrated in a study in which older adolescents undertaking a university apprenticeship calculated the environmental impact of their own lifestyle (including diet) and reported being surprised at the large contribution from food.^37^ Students perceived this as difficult to change, with only half of students willing to change their current dietary behavior to reduce the environmental impact.^37^ Adolescents reported competing priorities when making food choices and were unlikely to consider or value sustainability, unless this was directly prompted.

However, when questioned directly, some adolescents reported aspiring to reduce the environmental impact of their dietary choices.^31^^,^^34^^,^^37–39^ The studies in which environmental sustainability was valued most highly when making food choices were among populations with predominantly older adolescents in Europe or among rural indigenous communities in Alaska, the Pacific Islands, or the Caribbean.^31^^,^^35^^,^^40^^,^^41^ Adolescents who value sustainable eating have been identified from interviews and surveys as those from higher socioeconomic backgrounds who have received previous education about sustainable diets, have family and/or social support in maintaining sustainable dietary behaviors, are “leftist” (in a political sense), and hold complementary values (eg, health and animal rights).^42^^,^^44^ Some adolescents perceived sustainable dietary habits to be more “feminine”;^43^ however, there was greater interest from adolescent boys in Australia to prioritize learning about sustainable diets and food waste.^33^^,^^42^^,^^44^

Some aspects of sustainable diets were valued more highly or were more likely to be adopted by adolescents. Most commonly mentioned by adolescents, and in a variety of contexts, was an aspiration to support local producers (regardless of the context).^28^^,^^29^^,^^31^^,^^33^^,^^37^^,^^41^^,^^44^ In the Caribbean and New Caledonia, adolescents reported valuing the consumption of local cultural and traditional foods for sustainability.^29^^,^^31^ Other valued behaviors of sustainable diets among those sampled from a setting involving previous nutrition, environmental or agricultural education included consideration of animal welfare,^33^^,^^41^ reducing food packaging,^37^ and reducing food waste.^44^ However, there was particular disinterest and reluctance reported from older adolescents in Europe toward restricting their consumption of animal-sourced foods.^24^^,^^37^^,^^41^ These types of sustainable dietary behaviors most valued, such as consuming locally produced products and reducing packing and waste appear to be largely consistent for adolescents across countries, although the broader consideration of sustainability when making food choices appears to be depend on the context of the adolescent, their previous nutrition- or environment-related education, and competing priorities.

#### Perceived barriers to and enablers of adhering to sustainable diets

Finally, when considering the adoption or maintenance of sustainable diets, barriers to and enablers of sustainable-food consumption were explored (Table 2). The most commonly mentioned perceived barrier to consuming sustainable foods was cost, particularly by adolescents from lower socioeconomic backgrounds.^28^^,^^30^^,^^42^ Other barriers include unappealing taste, appearance or smell of “sustainable” food items (particularly vegetarian foods such as vegetarian lasagna).^24^^,^^25^^,^^38^^,^^42^ Geographical limitations, such as climate or land availability affecting the ability to grow or purchase local and organic products, were also mentioned as barriers to consuming sustainable foods.^29^^,^^30^

**Table 2.** Perceived barriers to and enablers of adolescents consuming a sustainable diet

Perceived barriers	Perceived enablers
Increased cost of sustainable foods Unappealing taste, appearance, or smell of sustainable foods Inability to grow or purchase locally produced foods Lack of knowledge regarding sustainable diets Distrust of product labels or claims Conflicting social norms	Access to food gardens Availability of sustainable foods and foods with limited packaging Appealing presentation of sustainable foods Clear and informative sustainability food labels Education regarding sustainable diets Supportive social norms

Adolescents reported a lack of understanding of sustainable diets and distrust of sustainability-related claims from fast-food outlets regarding the quality or source of ingredients, making it difficult to make informed food choices.^25^ Additionally, behaviors conflicting with personal and/or group norms were noted as barriers to adopting sustainable dietary habits. Examples of this include peer norms supporting the disposal of unfinished food (which may conflict with personal or family norms),^36^ a negative personal disposition toward adopting a vegetarian or vegan lifestyle,^42^ or the personal or group perception that plant-based foods are only for those that assume a vegetarian or vegan “identity.”^24^^,^^25^^,^^35^^,^^37^

Facilitators of sustainable dietary food behaviors were observed in numerous instances when adolescents made food choices. When sourcing food, access to fresh produce in community food gardens (local, home, or relatives’) was perceived as a facilitator and coping strategy to address the high costs of commercial organic produce.^30^ Simple dietary changes or swaps, or changes that required minimal cognitive choice (eg, change in the supply chain and availability of products) were mentioned as facilitators of future sustainable dietary habits.^35^^,^^37^ These include reduced meat availability and/or increase in plant-based options, increased availability of organic and/or locally produced foods, and reducing optional packaged foods within existing food systems (eg, school canteens).^35^ Appealing presentation and naming vegetarian alternatives in a way that normalized sustainable options (eg, a label of “lasagna” rather than “vegetarian lasagna”) also promoted simple dietary changes or swaps by reducing the perceived change in diet or diet identity for adolescents.^28^^,^^35^^,^^37^

Adolescents also reported that purchasing sustainable foods may be facilitated by easy-to-understand labels that indicate the product’s environmental impact and/or other benefits (eg, health, social equity, animal rights).^28^^,^^34^^,^^35^ This helped increase adolescents’ awareness of and attitudes toward environmental sustainability and the potential impact of their choices.^34^ Furthermore, previous education on diet-related sustainability, complementary ethical or moral values (eg, health, caring for nature, animal rights), and community norms in some settings were perceived as facilitators of environmentally sustainable-food choices, specifically in Europe among older adolescents who reported having some responsibility for their own diet.^24^^,^^31^^,^^42^ Promoting and influencing social norms of sustainable eating among personal networks was particularly noted by adolescents who had chosen to adopt sustainable dietary habits. They used strategies such as sharing personal beliefs, promoting and influencing social norms of sustainable eating among personal networks, and independence in food preparation to maintain their sustainable-food behaviours.^42^

### Interventions to improve the sustainability of adolescents’ diets

#### Intervention study characteristics

Included studies (n = 16) reporting on interventions to improve the environmental sustainability of adolescents’ diets were across different geographical and social contexts, and mostly (n = 11) published after 2015. Studies were predominantly from Europe (n = 8), North and Middle America (n = 6), and the United Kingdom (n = 1), with 1 study including participants from both the United Kingdom and Italy. Within and between these countries, the socioeconomic demographics of participants varied (Table S3). Most interventions (n = 14) were in the context of educational institutions. In terms of age, there was variation between studies with narrow (1–2 year)^26^^,^^40^^,^^45–47^ and broad (>3 year) age ranges.^48–50^ Some interventions targeted younger children (<13 years old) as well as adolescents,^38^^,^^51–53^ and others also targeting young adults (>18 years old).^31^^,^^37^^,^^41^^,^^52^

Interventions were underpinned by a theoretical framework of behavior change theory in 6 studies. Most interventions were reported as case studies (n = 8); some sources reported a pre-post study design (n = 6; n = 2 with a control group), and there was 1 cross-sectional study and 1 randomized control trial. There was variation in the framework used between studies; however, most acknowledged aspects of knowledge and motivation in driving change in attitudes and behavior.

#### Intervention types and facets of targeted sustainable diets

**Figure 2.**
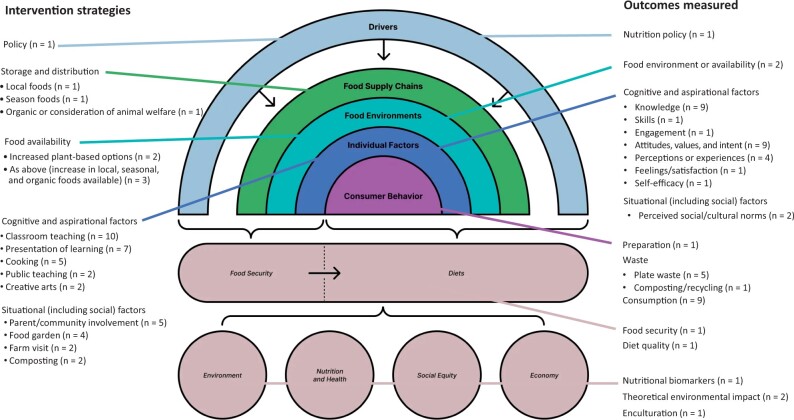
Intervention strategies and outcomes evaluated from the 16 included studies, mapped across the food system framework^6^

Several intervention types have been used to improve the sustainability of adolescents’ diets. Most (n = 11) are multifaceted, using more than 1 type of intervention. The most common interventions reported in the articles included in this review were classroom teaching (n = 10), adolescents’ presentation of learnings (n = 7), food availability (n = 5), cooking (n = 5), and parent and community engagement (n = 5). Less frequently reported intervention types included food gardens (n = 4), composting (n = 2), farm trips (n = 2), creative arts (n = 2), packaging and waste reduction (n = 1), public teaching (n = 2), and implementation of policy (n = 1). These types of interventions have been mapped across the food system and categorized according to the relevant component of the food system targeted, as displayed in Figure 2 (note that due to their interconnected nature, types of interventions addressing cognitive and aspirational individual factors have been listed together in the figure).

Of those interventions that were uni-faceted, most were related to food availability in terms of a change in the types of foods provided for adolescents.^38^^,^^51^ There was a variety of facets of sustainable diets targeted in the interventions, and all interventions addressed several principles of sustainable diets each, as defined by the FAO.^1^ Most interventions included aspects addressing the promotion of whole (ie, minimally processed) foods,^26^^,^^31^^,^^35^^,^^38^^,^^46–51^ reducing the environmental impact or resource demand (eg, GHGE),^26^^,^^31^^,^^35^^,^^37^^,^^38^^,^^40^^,^^41^^,^^46–52^ and reducing food waste or loss.^26^^,^^35^^,^^38^^,^^45^^,^^47–53^ Other principles that occurred commonly were increasing plant-based foods, reducing animal sourced foods (particularly for interventions of food availability),^26^^,^^31^^,^^35^^,^^38^^,^^40^^,^^47–51^ respecting local cultural practices of food sources production and consumption (particularly for those in lower socioeconomic or specific population settings),^31^^,^^35^^,^^38^^,^^40^^,^^41^^,^^47^^,^^48^^,^^50^ and increasing accessibility and desirability of sustainable foods.^31^^,^^35^^,^^38^^,^^40^^,^^48^^,^^50^^,^^51^^,^^53^ Principles relating to energy and nutrient adequacy,^26^^,^^38^^,^^41^^,^^45^^,^^46^^,^^53^ minimizing the use of antibiotics and hormones (organic meats),^41^^,^^48–51^ and food safety were less common.^26^

Some patterns were observed between the intervention type and facet of sustainable diets that was targeted. Interventions involving change to the food supply or food environment (ie, procurement or availability) addressed the health aspects of sustainability (ie, promotion of whole foods, increased fruit and vegetables, and decreased animal-sourced foods by promotion of vegetarian meals), as well as sociocultural aspects (accessibility and desirability) and environmental (ie, reduction in GHGE and waste).^35^^,^^38^^,^^40^^,^^50^^,^^51^^,^^53^ None of the intervention studies specifically addressed economic factors or affordability of foods as a sustainability outcome.

Teaching interventions varied in the principles of sustainability addressed; however, they often included several environmental principles (eg, GHGE, biodiversity, organics, waste).^26^^,^^31^^,^^37^^,^^40^^,^^41^^,^^45–52^ Those studies including agricultural elements of food gardens, farm trips, or composting also addressed multiple environmental principles of sustainable diets.^48–51^ Thus, the connected nature of the principles of sustainable diets facilitated addressing multiple aspects, regardless of the intervention type.

#### Methods used to assess intervention effectiveness

The methods used and outcomes measured to evaluate interventions varied depending on the type and aim of the intervention. The outcomes measured across the food system are displayed in Figure 2. Studies that involved teaching as an intervention evaluated participants’ cognitive behavior and consumer behavior quantitatively. Evaluation tools used to assess understanding included a knowledge questionnaire^45^^,^^48^^,^^52^ or grades of assessment,^46^ and intake was assessed using a food intake diary.^40^^,^^45^^,^^48^ Qualitative data on engagement in the teaching intervention, and knowledge and attitudes related to sustainable diets were collected through audio recordings of teaching sessions,^26^^,^^37^ group discussions,^31^^,^^37^^,^^41^ surveys,^40^^,^^47^^,^^50^ or content analysis of assessment items.^26^^,^^37^^,^^41^^,^^47^

Studies that involved changes through food procurement or availability evaluated factors relating to consumer behavior, individual factors, and/or the food environment. Evaluation methods included quantitative measuring of plate waste^38^^,^^47^^,^^51^ or mealtime attendance,^53^ and also food satisfaction surveys^38^ or food environment assessments.^51^ Some studies also used focus groups or interviews to collect qualitative data on the experiences and perceptions of adolescents^38^^,^^48^ or staff^38^^,^^47^^,^^50^^,^^51^ regarding the intervention targeting food procurement or availability. Thus, most intervention studies evaluated the effectiveness of the intervention on food system sustainability by assessing the impact on consumer behavior. Few studies evaluated the nutritional or environmental outcomes.

#### Effectiveness of interventions

Overall, regardless of the type of intervention or the facet or sustainable diets that was targeted, most interventions were successful in improving the sustainability of adolescents’ diets across key aspects of the food systems framework (Table 3). For those that involved classroom or public teaching only, there was minimal change in the attitudes, knowledge, or behavior of students relating to sustainable eating.^52^ However, knowledge increased when the intervention included problem- or activity-based learning techniques, such as group discussions with an educator’s feedback,^37^^,^^41^^,^^46^ cooking classes,^26^^,^^40^^,^^41^^,^^49^ using creative arts in teaching and learning,^31^^,^^47^ calculations of personal environmental impact,^37^ or providing education to parents.^45^ Students often provided positive feedback after these more engaging learning styles.^37^ Behavior change and positive attitudes toward sustainable eating increased further with adjunct interventions targeting food availability changes,^40^^,^^50^^,^^51^ adolescents’ presentation of their learnings to others (eg, to peers, the public, or parents),^40^^,^^41^^,^^47–49^ and parent or community involvement.^40^^,^^48–50^

**Table 3.** Effects of interventions targeting sustainable diets among adolescents

Intervention type	**Observed change in individual factors** ^a^	**Observed change in consumer behavior** ^b^	Barriers to intervention effectiveness	Facilitators of intervention effectiveness
Teaching	+	–	Difficulty tailoring delivery of education to large and/or diverse range of pupils Lack of time Lack of resources	Flexibility with education delivery mode Stakeholder support Engaging teaching style (eg, practical, creative, problem solving) Peer, family, or community involvement
Food procurement and/or education	–	+	Lack of time Lack of resources Lack of required skills	Consideration of cultural acceptability Consideration of taste preferences Gradual changes in food availability
Creative arts	+	+		Peer involvement
Agricultural exposure	+	+	Lack of resources	Peer or community involvement
Multicomponent	+	+	As above (depending on included strategies) Difficulty tailoring delivery of intervention to large and/or diverse range of pupils (limited scalability)	As above (depending on included strategies)

a Individual factors include cognitive understanding and aspirational values.^6^

b Consumer behaviors include purchasing and consumption of food.

*Symbols*: +, positive change observed as an outcome of the intervention; -, negative or no change observed as an outcome of the intervention.

Two studies focused on changes in the food supply, without educational or promotional interventions, and these studies had relatively lower effectiveness.^53^^,^^54^ One intervention, the OPTIMAT study, conducted in a culturally diverse area of Sweden, entailed a change to the foods available from the canteen menu (from which all students receive their food during school hours), with no supportive education, promotion, or involvement with the adolescents or school community in the design of the program.^38^ This study showed that a 4-week menu with a (theoretical) 40% reduction in GHGE, 14% reduction in cost, meeting greater than 97% of all nutrient requirements, and designed to be similar to existing menus can be implemented with no significant difference in plate, serving, or consumption waste from baseline, or change in students’ satisfaction of the meals.^38^ However, the level of satisfaction of school meals remained low, and it is unclear how other environmental impact measures may have changed as a result of this project (eg, use of plastics or packaging waste; water and land use). A similar study in Finland introduced a mandatory vegetarian-only day each week to the (optional) national school lunch program for at least 6 months, with no adjunct interventions.^53^ This was in the context of some local political debates and public opposition of the idea of food restriction for children at school. The intervention initially had a negative effect on consumption, as evidenced by a decrease in participation in school lunches by 19%, and increase in plate waste by 40% for lower-secondary students and 89% for upper-secondary students 11 weeks after the initiation of the intervention.^53^ However, after 6 months, there was no change in plate waste from baseline, and consumption of vegetarian meals increased in lower-secondary schools.^53^ Changes in the amount of food produced, environmental impact of the changed menu, or students’ satisfaction were not measured.

Two studies integrated creative arts uniquely into their respective interventions.^31^^,^^47^ One study based in the Caribbean (delivered online across the United Kingdom and Caribbean), used the composition of a song to allow older adolescent participants to creatively engage and share their understandings of climate change and food system.^31^ This allowed participants to process the content of the discussion deeply and creatively, and communicate their relevant values and beliefs through a culturally appropriate mode at a large-scale public event.^31^ With a younger and larger group of adolescents in the United States, students’ designed creative posters to share their learning with older students regarding food waste, and displayed these around the school cafeteria.^47^ This resulted in a significant increase in students’ knowledge of and attitudes toward food waste, and a decrease in food waste at the school.

All studies that included exposure to agricultural aspects of the food system in the intervention were part of multicomponent interventions.^48–50^ Interventions exposing students to the agricultural aspects of sustainable foods through food gardens, composting, or farm visits were commonly effective in improving students’ awareness of the environmental impact of food systems and changes in dietary habits.^48^^,^^49^ One study in a single private school in Mexico showed that by having additional lessons in a school food garden growing sustainable foods, students’ fruit and vegetable consumption increased by an average of 25 g/d, and some students had subsequently initiated gardens at their home.^48^ This was similar to the results reported from a project with a small community youth group in Canada in which participants were involved in managing a food garden.^49^ Most participants involved in food gardens reported an increase in fruit and vegetable intake, an increase in understanding of food systems and production (global food production, and organic and local foods), and improvement of food (and general) waste practices of the youths and local community resulting in less litter.^49^ In programs involving food gardens, there were broader benefits in that participating adolescents often enjoyed the programs and reported that they strengthened community relationships, gained skills, improved food security, and improved self-esteem.^48^^,^^49^ However, a large multicomponent intervention involving food gardens in 24 schools in the United Kingdom achieved no changes in attitudes toward or behaviors aligning with sustainable-food practices, although positive attitudes toward sustainable food were associated with sustainable dietary habits.^50^

Interventions that involved multiple components across the food system were more likely to be effective in improving sustainable dietary behaviors. Even without changes to the food supply, studies that had multiple components, particularly with hands-on learning in food gardens, cooking, or creative arts in conjunction with education, were effective in improving sustainable food–related knowledge up to 3 months after the intervention.^31^^,^^41^^,^^45^^,^^47^ Changes in dietary intake were reported with changes in the foods supply, as seen from the study by Bersamin et al,^40^ in which 3 strategies (education, increased availability of locally produced fish, and community involvement) together over 9 months resulted in an increase in local fish consumption. This change in consumer behavior occurred despite no observed difference in attitudes toward or perceptions of environmentally sustainable foods between the control and intervention groups.^40^ An exception to this was seen in an older study from 2012 by Jones et al^50^ in the United Kingdom, which involved a large-scale, multicomponent program, Food for Life Partnership, in 24 secondary schools. The program entailed school food procurement, availability and policy interventions, food gardens, cooking classes, and farm links.^50^ After 18–24 months of the intervention, there was no significant increase in fruit and vegetable consumption or change in attitudes toward sustainable foods.^50^ The authors of this study suggested the breadth of their target population may have limited the depth of their intervention’s impact due to the difficulty in tailoring interventions for each school’s population of students and staff, and resources.^50^ Overall, multicomponent interventions with close engagement by participants (either in the design of the intervention, hands-on education, or through creative arts) were effective in improving the knowledge, attitudes, and/or behaviors of adolescents regarding sustainable diets.

#### Barriers to and enablers of intervention implementation

Some studies reported on barriers to and facilitators of implementing the intervention. Staff reported challenges in the implementation of changes to food procurement or availability, including time and financial constraints, challenges in coping with change or with acquiring new skills, burdensome paperwork, and poor communication or engagement with stakeholders.^35^^,^^38^^,^^47^^,^^50^ Similarly, barriers reported by school staff implementing educational interventions included time constraints, difficulty tailoring information to large and/or diverse target populations, and additional administrative work.^47^

Common facilitators of programs largely related to social, cultural, or community aspects of interventions. Well-planned interventions, with adequate time and consultation with key stakeholders, were found to assist in overcoming barriers and enabled the delivery of interventions.^38^ Promotional strategies such as highlighting environmental benefits, avoiding labels referring to discrete “diet identities” (eg, vegetarian), appealing to personal or cultural taste preferences, and using familiar foods with gradual changes to the menu resulted in more positive responses to food procurement and availability interventions by adolescents.^38^ Teachers reported flexibility with curriculum delivery and access to support and training, as facilitators to successful delivery of an educational intervention with a relatively large cohort of participants.^47^ Overall, the interventions that were appropriately planned with relevant stakeholders and resources facilitated more effective outcomes.

## DISCUSSION

From this scoping review, the perceptions of and interventions targeting adolescents regarding sustainable diets were reviewed from 28 included sources. Overall, the results indicate most adolescents have limited understanding of what constitutes a sustainable diet, and sustainability is not a primary factor when they make food-related decisions. Interventions aimed at improving the sustainability of adolescents’ diets were primarily delivered to improve the individual’s cognitive knowledge, understanding, and aspirational value of sustainable diets, with some positive impact on the participant’s perceptions of sustainable diets. However, few studies have investigated the effectiveness of interventions on the dietary behavior of adolescents.

### The contexts of included studies, including food systems and adolescents’ role

The contexts in which adolescents’ perceptions of sustainable diets have been recorded and interventions have been implemented to improve dietary sustainability were predominantly in high-income countries. This finding reflects a potentially biased spectrum of political and economic contexts. An increase in political and economic interest in environmental sustainability (not only within the context of health and nutrition) is growing, particularly in Scandinavia and other westernized countries,^55^ reflecting most settings of the studies included in this review. Therefore, perceptions of environmentally sustainable diets may have been more positively acknowledged due to local social norms. The effectiveness of interventions may have been greater due to stakeholders involved in the interventions (eg, schoolteachers and principals) acknowledging and accepting that dietary choices have environmental impacts.^35^ This may also have led to greater likelihood of availability to the required resources for intervention implementation.

This scoping review has identified a gap in the literature regarding adolescents’ perceptions of sustainable diets and interventions to promote sustainable diets in low- and middle-income countries. These countries may have food systems with alternate characteristics (eg, different food production, transport, marketing, cooking and consumption methods) and different priority principles of sustainable diets to target (eg, adolescent breastfeeding, food safety, adequate nutrient intake, and safe water access) and, therefore, require adaption of future interventions to support adolescents’ healthy and sustainable diets in these contexts.^1^^,^^56^

Overall, the characteristics of the food systems and food environments in which interventions were implemented were similar. In the context of Sweden or Finland, students were provided meals from a rotating menu each day at school. In countries where school meals are not provided (ie, children bring packed food from home or purchase from local shops) or optionally are accessed from school tuckshops (ie, a small shop, located near a school, that sells snack food; as commonly occurs in Australia), methods used in this intervention would need to be adapted to the context of the local food system, particularly considering how, where, and by whom foods are procured and prepared. Evidence for environmentally sustainable food-system changes in these settings in which parents and/or adolescents are responsible for school meals are lacking.

Furthermore, there were limited reports on the degree of autonomy or responsibility that each study’s participants held in terms of their food choices. An adolescent’s responsibility for food choices and food literacy varies greatly depending on their sociocultural setting, age, sex, and household role.^13^ For example, in settings like that of the Colombo et al study,^35^^,^^38^ the food environment was such that participants were provided a set meal at schools and had no responsibility for the preparation of this, or were involved in choosing the meal to consume. This environment and the adolescent’s autonomy and agency over food choices would be different in an Australian scenario in which the adolescent has a choice to prepare and bring their own lunch to school, purchase food from a canteen, or their parent may take all responsibility for their child’s food choices. Consideration of a target population’s context and adolescents’ roles and responsibilities in food-related behaviors are important when designing interventions.^13^

### Adolescents’ cognitive understanding of sustainable diets

Despite an awareness of broader environmental issues,^12^ adolescents’ understanding of the sustainability of diets was generally poor, with much greater knowledge reported of the health, rather than environmental, impacts of diets. Adolescents’ understanding of healthy eating has previously been reported as often being quite accurate,^23^ likely a result of substantial previous research, education, and nutrition interventions (including policy) targeting school food environments. Given this effectiveness, future investment in education and strategies in school food environments to promote sustainable dietary choices should be supported.

Where there was some understanding of environmental sustainability of diets, it was perceived that the environmental sustainability of a diet is determined by factors out of control of the adolescent consumer, such as production, processing, and transport methods, and the impact of personal food choice was not well understood. An Australian study reported that among adolescents, greater knowledge of sustainability and the belief that individuals and the community have responsibility and the ability to undertake pro-environmental behaviors result in more engagement in environmentally sustainable actions.^57^ On the other hand, belief that the government holds greater responsibility for environmental sustainability was associated with less engagement in pro-environmental behavior among older youth (<24 years of age).^57^ Thus, these findings indicate it is challenging for adolescents to understand the complexity and interconnectedness of the food system and the role that consumers play in it. Evidence from other studies indicates there is often a gap between knowledge of what entails healthy and sustainable food choices and pro-environment behavior change. However, there still remains a stepwise association between acquiring cognitive knowledge and understanding, a change in attitudes, and then a change in behaviour.^58^^,^^59^

### Aspects of sustainable diets addressed

The predominant principles of sustainable diets (as defined by the FAO and the World Health Organization^1^) that were targeted in the interventions included in this review were those related to the healthiness and environmental aspects of diets. Other principles defined by the FAO and the World Health Organization, such as food safety, breastfeeding, and gender equity, were not studied; this may be explained by the predominant setting of high-income countries in which the agricultural and commercial production of foods, or breastfeeding, are not typical activities for the majority of adolescents. From a food system perspective, it is important to note that behaviors supporting a nutritious diet may also support environmentally sustainable dietary behaviors, depending on context.^60^ Given the abundance of previous interventions to support healthy diets among children and adolescents, interventions included in this review often used methods similar to existing school nutrition interventions targeting healthy eating, and focused on the healthiness of diets as well as the environmental aspects (eg, promoting plant-based foods).^61^ The most commonly targeted environmental principle of sustainable diets was to reduce natural resource use and emissions through a decrease in consumption of animal-sourced foods. This aligns with the EAT-Lancet framework suggesting that a global reduction in animal-sourced foods (especially in high-income countries), increase in consumption of plant-based foods to meet (and not exceed) energy requirements, and a reduction in food waste is needed to for sustainable diets.^62^ Interestingly, no studies addressed the overconsumption of foods; they only addressing food waste. However, interventions targeting overconsumption may be more likely to have been framed from a health (only), rather than environmental, perspective and, therefore, were excluded from the review.

### Adolescents’ aspirational value of sustainable diets

In addition to knowledge and understanding of sustainable diets and the consumer’s role, adolescents’ perceptions or aspirational value of sustainable diets can also motivate behavior change.^58^^,^^63^ Evidence from the studies in this review suggests that there may be 2 distinct groups of adolescents who have greater consideration of the environmental impact of their dietary choices: older adolescents from higher socioeconomic backgrounds in an educational setting (eg, urban schools in Scandinavia) and those of any age from rural or indigenous communities. Previous studies have supported this idea^64–66^ and identified a decline in pro-environmental attitudes among adolescents aged 15–16 years, a time during which adolescents’ autonomy increases and consideration of others decreases.^67^ However, there is consistency in the literature suggesting that adolescents and adults who value factors such as nutrition, care for nature, and animal protection are more likely to value the environmental impact of dietary choices. Furthermore, given the interconnectedness of these aspects in the principles of sustainable diets, there is potential to piggyback or adapt interventions that target more commonly valued aspects of health and taste, or care for nature, that may align with the promotion of sustainable diets.^68^^,^^69^ Adolescents who value the environment should be empowered and engaged in understanding and addressing sustainability issues, and making behavioral changes.^12^

### Adolescents’ perceived barriers to and facilitators of sustainable diets

Factors perceived by adolescents as barriers to or enablers of sustainable food consumption included appeal, accessibility, and social norms. Sensory appeal (eg, taste, smell, appearance) of food is a known predominant factor of food choice among adolescents^9^ and should be considered when promoting sustainable foods. Cost has been reported as a perceived barrier to healthy and sustainable eating, particularly for those of lower socioeconomic status and with lower food literacy.^57^ Even if a healthy and sustainable diet were more affordable, it requires food and nutrition literacy to purchase, prepare, and store sustainable foods appropriately.^70^ Accessibility to products or gardens to grow or from which to purchase sustainable foods is also a reported factor that may be supported in schools through food supply or school gardens.^71^^,^^72^ Labels indicating sustainability of products have had mixed success as a facilitator of sustainable choices, because some consumers are untrusting or lack understanding of how to interpret or use these labels.^73–75^ Importantly among adolescents, social networks (including both peers and parents) influence food choices^9^^,^^75^ and, therefore, community support and a shift in social norms are needed to support adolescents’ sustainable food choices.^72^ For example, in a targeted intervention, the use of visual posters, information booklets, marketing in newspapers and social media, as well as publicity at community events have been shown to effectively shift social norms in Australia regarding underage alcohol consumption.^76^ Interventions that consider appeal, affordability, and social norms are needed to address adolescents’ perceived barriers to sustainable food choices.^77^

### Characteristics and effectiveness of interventions to support sustainable diets among adolescents

The evidence indicates it is recommended to tailor interventions to improve the environmental sustainability of adolescents’ food choices to the specific target population (ie, individual school or community group, age, and setting) and to consider collaboration of adolescents within the intervention design.^56^^,^^78–80^ It is worth noting that the observed increase in effectiveness of strategies when tailored to a specific target population limits the scalability of interventions and is often in conflict with the scale of change that is needed. Interventions targeting upstream components of the food system (eg, food supply, food environment), such as school feeding programs, may present opportunities for more scalable change.^81^

Although multicomponent interventions were most effective, across the food system, the interventions were largely targeting individual factors through education, and/or targeting the food environment through changes in food availability.^82^ Interestingly, the strategies of classroom education, peer or parent involvement, increased accessibility to sustainable foods, and food environment modifications are the same as those used to improve the nutritional quality of adolescents’ diets, which may reflect the recommendations and learning from previous healthy eating interventions in schools.^78^ Additional strategies to promote sustainable consumption at schools, including incorporation of vegetarian menu items, sourcing local and organic foods, promoting a reduction in food waste, composting, and use of food gardens, have been recommended,^71^ and it is noted that these strategies address a broader range of components within the food system (ie, food supply and waste management). There were minimal interventions in the literature targeting food-supply-chain interventions, and this may be attributable to the inclusion criteria specifying that the target adolescents must be considered consumers within the context of the food system for the study. There were also few interventions targeting broader drivers, such as policies; however, nutrition policies in schools have been reported to have reduced effectiveness due to the required resourcing for ongoing monitoring.^83^

Regarding adolescents’ individual factors and education, studies in this review focused on education provided in schools. Education and support for parents of adolescents may provide an another opportunity to promote and adopt sustainable diets through modelling and development of personal norms^74^^,^^77^; however, this was not observed in studies included in this review. A recent systematic review reported that healthy-nutrition education in schools, delivered with a family component and through the curriculum with teacher support, had no significant impact on consumer behavior.^84^ In accordance with previous research among adolescents, educational interventions with creative, experiential, or practical learning techniques may be more likely to result in a gain in nutrition- or food-related knowledge.^56^^,^^85^^,^^86^ Nutrition education through schools, including cooking or home economics classes, and school gardens, may improve nutritional knowledge, skills, and attitudes.^72^^,^^87–89^ However, there appears to remain a gap between acquiring food-related knowledge and implementing desirable dietary changes.^33^

Nutrition educators in schools are supportive of the inclusion of environmental sustainability in the curriculum, but they report requiring financial and technical support through schools and governing bodies.^90–92^ There may be opportunities to use technology in sustainable nutrition education for adolescents, like previous nutrition-promotion programs in schools using virtual realities, computer games, mobile apps, or photovoice techniques for engagement with students.^93–97^ The long-term effect of school nutrition programs is unclear, particularly for behavior change,^78^ and, similar to studies with adolescent participants, there is a lack of studies evaluating the food-choice outcomes of adult populations receiving nutrition education.^98^ However, there is growing support for the inclusion of sustainable food education in schools because there is potential to address concerns of both human health and planetary health in an engaging, consistent, and scalable manner.^72^^,^^97^^,^^99^

Interventions implemented among adolescents to support environmentally sustainable dietary changes through the food environment are similar to those for adults.^100^ However, studies among adults tend to less often restrict availability of animal-based foods and often focus more on use of food labels and prompts at the point of sale.^100^^,^^101^ Nudging strategies such as intentional product placements, verbal or written prompts, traffic-light-system labels (ie, green, yellow, red), or enhancing the convenience of nutritious menu options were reported, in a recent systematic review of healthy school food environment interventions, in food environments with limited, but positive, evidence of effectiveness to promote healthy food choices, and also among European adults to promote sustainable food choices.^77^^,^^102–104^ Interestingly, in the studies included in this scoping review, interventions in which there was a change in food supply and availability to support environmentally sustainable options of adolescents were not usually accompanied by supporting nudging strategies. However, protocols for future studies have been published that consider food-availability changes in schools with supporting nudging and school-wide interventions in Europe and Tanzania.^105^^,^^106^ Other studies have quantitatively evaluated the nutritional and environmental impacts of a planned school menu for optimal benefits^107–109^; however, these are yet to be implemented. Interventions that involved changes to menus or the food supply invoked initial negative responses by adolescents in some studies (indicated by increased plate waste and decreased meal satisfaction); however, this seemed to improve in later stages of the intervention.^78^ The long-term effectiveness of these types of interventions to influence dietary habits into adulthood, and their environmental and nutritional effects, are unclear and warrant further research.

Interventions were evaluated using measures similar to those used in other nutrition interventions. Most interventions’ evaluations included in this review measured components within the food system, rather than health or environmental outcomes. This may be due to the increased complexity of, and resources and time required for, evaluating distal outcomes such as environmental and nutritional impact, and these are recognized limitations of previous nutrition interventions with adolescents.^78^^,^^110^

Barriers such as lack of time and resource restriction were reported to inhibit the effectiveness of implementing interventions, whereas clear communication and good organization and functioning relationships between stakeholders facilitated effective interventions.^35^^,^^38^ These factors echo those reported for other nutrition and lifestyle behavior interventions that involved children and adolescents, as well as adults.^111^^,^^112^ Where feasible, future interventions should consider evaluating the longer term and broader dietary, environmental and nutritional outcomes of interventions, and collaborate closely with stakeholders, including adolescents, to promote effective design and implementation of interventions.

### Limitations

There are some limitations to this review that should be noted. First, studies were included only if they referred to environmental sustainability within the text or were framed within the context of sustainability. There are other studies that would likely report environmental benefits without specifically focusing on such an outcome. For example, studies targeting health or nutrition-related outcomes (eg, food literacy, fruit and vegetable intake, consumption of plant-based diets) or cost outcomes (eg, food waste^113^) were not included in this review. However, such studies may also provide insight into successful intervention methods for influencing food choices by adolescents and may provide additional relevant information about adolescents’ dietary behaviors. The interconnected nature of sustainable diet principles within the food system perpetuates the limitation that, arguably, any nutrition intervention that achieves shifts toward healthier diets may also improve the sustainability of the diet, depending on context.

Similarly, this review focused on adolescents to identify interventions tailored to this age group. Because of this specific focus, our review did not include research that consisted of population-wide interventions. However, although interventions in broader studies may not have included interventions tailored toward adolescents, some of these whole-of-population interventions may have been inclusive of adolescents and their methodology, and results may be valuable to consider when targeting adolescents.

## CONCLUSION

Overall, the environmental sustainability of adolescents’ diets is of growing interest, with multiple published studies in recent years reporting adolescents’ perceptions and interventions implemented to increase adolescents’ knowledge and value of sustainable eating, and to support environmental changes for them to be able to make environmentally sustainable food choices. This scoping review indicates that adolescents’ understanding of what constitutes sustainable eating is generally low, contributing to limited consideration of the environment when making food-related choices. Those who previously received education regarding environmental sustainability and the values of nature and health, or who were from a rural or indigenous community, were more likely to value environmentally sustainable food choices. Broad interventions that target adolescents’ cognitive understanding and aspiration to make sustainable food choices appear to improve adolescents’ attitudes toward sustainable food, without clear improvements in their dietary behavior. On the other hand, interventions that seek to alter the food availability for adolescents, improve the sustainability of adolescents’ dietary intake without clear improvements in their sustainable food-related knowledge or attitudes.

Overall, interventions resulting in positive changes to knowledge, attitudes, and (to a lesser extent) behaviors of sustainable diets among adolescents addressed multiple aspects of the food system and considered the specific target population. The long-term effect of these interventions remains unclear. More research is needed in diverse contexts with consideration of the extent to which adolescents have responsibility for their own food choices, such as in lower- and middle-income countries, and school environments using a lunchbox or tuckshop model. Future research would ideally monitor long-term outcomes.

## Supplementary Material

nuae052_Supplementary_Data
