# Supplementary material for: Toward Sustainable Diets—Interventions and Perceptions Among Adolescents: A Scoping Review
Source: Nutr Rev. 2024 May 29;83(2):e694–710. doi: 10.1093/nutrit/nuae052 (PMC11723159; doi:10.1093/nutrit/nuae052)
Supplement: nuae052_Supplementary_Data [file nuae052_supplementary_data.zip › nuae052_Supplementary_Data/Supplementary information A - Search strategy.docx]

## Supplementary Material - Appendix A: Search strategy

Search strategies consisted of five concepts, and were limited to publications from the past 10 years:

| Concept | Search terms |
| --- | --- |
| 1. Adolescent | - Adolescent - Secondary student - Secondary school student - Teen or youth - High school student |
| 1. Diet | - Diet habits - Feeding Behaviour (Food choice, Food Consumption, Food waste, Food literacy, preparation) - Food systems (Food environment) - Sustainable and healthy diets - Food |
| 1. Environmental sustainability | - Waste - Environmental sustainability - Environmental impact - Climate (change) - Climate-friendly - Sustainable - Carbon footprint - Environment - Ecosystem |
| 1. Intervention | - Intervention study - Strategy - Program - Education |
| 1. Perception | - Perception - Perspectives - Motivators - Knowledge - Understanding, comprehension - Values - Attitude |

For example, in PubMed; ((((((((understanding[MeSH Terms]) OR (perception[Title/Abstract])) OR (perspective*[Title/Abstract])) OR (knowledge[Title/Abstract])) OR (understanding[Title/Abstract])) OR ((((intervention*[Title/Abstract]) OR (strateg*[Title/Abstract])) OR (program*[Title/Abstract])) OR (educat*[Title/Abstract]))) AND (“high school student” OR "adolescent"[MeSH Terms] OR "adolescen*"[Title/Abstract] OR "secondary school student"[Title/Abstract] OR "secondary student"[Title/Abstract] OR "youth"[Title/Abstract] OR "teen*"[Title/Abstract])) AND ((((((((((behavior, feeding[MeSH Terms]) OR (diet habits[MeSH Terms])) OR (diet* habit[Title/Abstract])) OR (food choice[Title/Abstract])) OR (food waste[Title/Abstract])) OR (food literacy[Title/Abstract])) OR (food preparation[Title/Abstract])) OR (food system[Title/Abstract])) OR (food environment[Title/Abstract])) OR (food[MeSH Terms]) AND (y_10[Filter]) AND (y_10[Filter]))) AND ((((((((environmental impact[MeSH Terms]) OR (environmental sustainability[Title/Abstract])) OR (sustainab*[Title/Abstract])) OR (climate[Title/Abstract])) OR (waste[Title/Abstract])) OR (environment[MeSH Terms])) OR (carbon footprint[MeSH Terms])) OR (ecosystem[MeSH Terms]) AND (y_10[Filter]))
