# Supplementary material for: Toward Sustainable Diets—Interventions and Perceptions Among Adolescents: A Scoping Review
Source: Nutr Rev. 2024 May 29;83(2):e694–710. doi: 10.1093/nutrit/nuae052 (PMC11723159; doi:10.1093/nutrit/nuae052)
Supplement: nuae052_Supplementary_Data [file nuae052_supplementary_data.zip › nuae052_Supplementary_Data/Supplementary information B - Summary table perceptionsV3.docx]

## Supplementary information - Appendix C: Data extraction table - adolescents’ perceptions of sustainable diets

| Citation | Context (country and setting) | Participants (age, number) | Understanding, value or barriers/enablers | Study aim | Theory or framework | Methods | Key findings | Conclusion/recommendations | Limitations |
| --- | --- | --- | --- | --- | --- | --- | --- | --- | --- |
| Bailey, Prichard, Drummond and Drummond, 2022^1^ | Australia High school students studying home economics, physical education or involved in elite athlete school programs at high schools (2 X public and 1 X private) in Adelaide. Schools across 3 socioeconomic tertiles were included. | Adolescents, average age of 15 years (n=27; 17 girls, 10 boys) | Understanding Barriers/enablers | The study aimed to explore Adolescent's beliefs and perceptions towards healthy eating behaviour, and differences across SES. | Symbolic and moral boundary work Social constructionist | Participants were recruited after approaching local schools, through teachers. Data was collected using focus group discussions (n=7) with an interview guide. The groups were <45mins in duration and audio recorded. Transcriptions of audio recordings were analysed thematically. The research team collaborated during the coding process to reduce bias and included a peer debriefing session. | Three main themes were identified; 1) beliefs and perceptions of non/healthy, 2) moral judgement of family and foods (self-identity, acceptance of friendship, comparison and family members), and 3) priority on ethical eating and sustainability. **Ethical eating was was defined as purchasing locally sourced and organic, with consideration of sustainability and animal welfare, some mentioned vegan/vegetarian, or purchasing free-range meat.** Some participants checked labels for locality or sustainability claims. **Barriers were cost** (especially for participants from low socioeconomic backgrounds) however this was **overcome by using gardens** from home, the community or relatives. There was a reported distrust of fast-food claims/advertisements. | Further research should look at how these perceptions translate into behaviour. | Adelaide only (not representative of Australia), potential for selection bias (with teachers responsible for recruitment and nomination of participants) Focus was of the study was on 'healthy' eating, with consideration of **ethical** eating |
| Bersamin, Izumi, Nu, O'Brien D and Paschall, 2019^2^ | Alaska Remote, native Alaskan communities (low income, off road, predominantly local/traditional foods, distributed independently, at risk of food insecurity - schools procure shelf-stable/processed foods via plane to supplement locally grown produce) | Middle and high school students, average age 14yo (n=76) | Value | The study aimed to evaluate a food systems intervention on diet quality, intake and attitudes and beliefs. | A framework was developed with the local community, based on Social Cognitive Theory which identified 9 main themes (including behavioural capability, collective efficacy, outcome expectations and self-efficacy). | All students at the local middle and high schools were invited to participate. Self-reported data was collected pre- and post- intervention (baseline, at 4 months and at 9 months after commencement, across one academic year). Data collected included fish intake (measured using a 24hr recall and blood biomarker), diet quality (Healthy Eating Index) and enculturation. A survey was conducted following the study to explore attitudes and beliefs regarding health and wellness (perceptions of salmon benefits, impact of eating traditional foods, attitude towards environmental impact of food, attitudes towards and value of having required food skills). | Differences in **attitudes towards impact of food choice on the environment were moderate (2.2/4) at baseline, and increased with time during the intervention**. | Local people had some consideration of the environmental impact of food choices, and this increased with regular exposure to locally produced foods and education. | The quantified amount that attitudes towards impact of food choice on the environment was not reported. |
| Collins, Galli, Patrizi and Pulselli, 2018^3^ | UK and Italy Cardiff University (UK), School of Geography and Planning, specifically students in a postgraduate unit relating to research measures of environmental impact. University of Siena (Italy) offered a sustainability course for all students and employees, and high school apprentices. The course focusses on environmental consequences of consumption. In this study, the high school students were in their third or fifth year at a local Scientific High School (general education with scientific focus), or third year at a Technical High School (specialising in laboratory teaching) | Post graduate (n=20) and high school (n=31; 74% male) students. | Value Barriers/enablers | The study aimed to assess the ecological footprint of students, and its calculation as an educational tool. | Not specified | Participants were recruited through the university and the intervention was included as part of their studies. Data collected included the ecological footprint (as global hectares derived from National Footprint Accounts, Consumption Land-Use Matrix (eg. carbon, grazing land, cropland etc) and National Footprint Accounts by consumption category at a geographical level) for each student as a reflection of their current lifestyle and with consideration of potential lifestyle changes. Results were compared between age groups, across categories, and before and after theoretical lifestyle changes. Data was also collected from classroom discussions. | High schoolers were **surprised about the large contribution from 'food', and perceived this as difficult to change** (suggested that a change was needed in the **supply chain**, not consumer choice). **Only half of students were willing to change their diet to reduce the ecological footprint.** Suggested changes included decrease meat consumption, decrease packaging, swap to organic and/or locally produced foods. There was **reluctance from participants to change to strict vegetarian or vegan diets.** | Older adolescents may underestimate the environmental impact of their diets. Barriers to more sustainable diets include restrictive diets (avoiding meat), whereas smaller changes were viewed as more realistic. | No clear methodology for data collected during classroom discussions. No report on age of high-school apprentices, however likely older, well-educated adolescents. |
| Colombo, Elinder, Patterson, Parlesak, Lindroos and Andermo, 2021^4^ | Sweden Stockholm schools (Grade 0-9), 52% of local population were not Swedish, high proportion of parents without tertiary education OPTIMAT study | Students in grade 5 and 8 (n= 29) | Understanding Value Barriers/enablers | Exploration of students experiences, barriers and facilitators 2' OPTIMAT intervention | Not specified | Participants were recruited selectively through selection of names from class lists to invite 8 students per grade, per school with gender diversity.  Focus groups were conducted for a duration of 24-50mins. Topics included; general perceptions of school lunch, experiences receiving menu, perception of sustainable diet, barriers/enablers of increase plant based meals .  Analysis was completed with an inductive approach with transcriptions of the focus groups. Each transcript was read multiple times, and codes, subcategory and category groupings were developed by the research team. | **Perceived diet sustainability to be related with meat consumption and food waste, with recognition of health co-benefits.** Reported **valuing environmental impact of food** but difficulty making behaviour change. Sustainability was not reported as a factor when making food choices. Perceived barriers to consuming sustainable foods include negative peer perceptions, unappealing taste/appearance/smell/ of food, dislike of school meals in general, and using labels relating to diet identities (put off by vegetarian label). Perceived enablers of acceptance of sustainable meals include highlighting personal benefits of plant-based foods, careful naming (avoid 'vegetarian' labels), offering familiar dishes (eg. those that resemble meat-based dishes such as lasagne), increase exposure/normalisation, and gradual changes. | Disconnect between awareness and value of environmental impact of foods and dietary choices was noted. | Limited participation (40% did not participate) therefore likely some bias in responses. |
| Damen and Steenbekkers, 2022^5^ | Netherlands High school student living with parents. | Adolescents aged 15-18 years (n=20; 13 girls, 7 boys) | Understanding Value | The study aimed to assess adolescents choice of snack bars, and the extent of consideration of healthiness, naturalness, sustainability, and time, impact and effect have on this choice. | Not specified | Participants were recruited via social media and snowball sampling. Data was collected from semi structured individual interviews using a piloted interview guide. The interview included questions on perceptions of healthiness, naturalness and sustainability of snacks (in general, and in relation to specific stimuli products). The interviews were carried out virtually, and recorded. Transcripts were analysed and categorised based on the topics of healthiness, naturalness and sustainability. | Naturalness: 11/20 reported naturalness as a difficult concept however adolescents mentioned fruit, not processed, healthier as associated ideas.  Sustainability: **12/20 did not know what sustainability meant in context of food. Of these, most could identify some aspect, with time and prompting from stimuli (eg. packaging). When described as sustainable, this was explained as good for the environment, planet and climate.** Most often adolescents **reported sustainability is identified by statements on packaging, the type of packaging, if it is good for environment, if it is produced in the own country.** **13/20 spontaneously reported sustainability was not important to food products (3/20 for healthiness).** Healthiness: participants were aware of this concept and able to list attributes (with and without stimuli). Attributes included: low in sugar, fruit, nutrients, nuts, protein. | Most adolescents were unsure of the meaning of sustainability of foods and there was no mention of different types of foods/food groups. Using stimuli provided more detailed responses. | Prompts from interviewer and stimuli may have altered participants perceptions. There is limited description of the study population's context. |
| Derler, Berner, Grach, Posch and Seebacher, 2020^6^ | Austria High school students attending a polytechnical school, or nutrition and agriculture school | Secondary school students aged 15-19 years (n=117) | Understanding Value | The study aimed to enhance high school students competencies related to food, food technology and sustainability. | Problem-based learning: knowledge, understanding, applying | The method of recruitment is not discussed. Data collected includes dietary intake (using a pictorial and written food diary, and survey regarding food preferences) at baseline. | Students reported taste, health, animal welfare and appearance of food to be important, as well as **homemade, regional and seasonal foods. Vegetarianism and veganism were unimportant.** Students were challenged with applying **competing aspects of sustainability food (eg. cost, nutrition, environmental impact etc).** | Students appeared to value more 'human' aspects of sustainability either for themselves (taste, health, appearance) or the community (homemade, regional, season). | It is not reported clearly to what extent homemade, regional and seasonal food is important to participants. |
| Dornhoff, Hornschemeyer and Fiebelkorn, 2020^7^ | Germany Approximately 10 different schools in northwest Germany, in and around Osnabruk, where there is a high prevalence (13%) of vegetarians (as opposed to 4% of the German population). The different school consisted of Gymnasium (intensified general education), Realschule (extensive general education), and Hauptschule (basic general education). | Students in 10th grade, average age of 15.5 years (n=46; n=16 from Gymnasium, n=15 from Hauptschule, n=15 from Realschule). | Understanding | The study aimed to explore students perception of sustainable nutrition, in relation to definitions by FAO and van Koerber. | FAO and van Koerber definition of sustainable nutrition (5 dimensions) | Recruitment occurred through schools, by teachers. Students were recruited in order or response (but blinded to topic of sustainability). Individual interviews (40-113min in duration) using a pre-tested interview guide were conducted. Interviews consisted of 4 phases; 1) perception of sustainable nutrition (list 10 terms), 2) conceptions of dimensions of sustainable nutrition (provided), 3) conception of recommendations for sustainable nutrition (provided), 4) assumed connections between dimensions of and recommendations for sustainable nutrition. During the interviews, a stimuli product (banana with sticker) was used to prompt participants. Data was analysed from the transcription, using deductive and inductive approaches based on the theoretical dimension of sustainable nutrition. | Health was most commonly mentioned (159 statements), then **ecological (77)**, social and economic, (37 and 23), and cultural aspects less frequently (7) (potentially due to current teaching structure). Most participants only mentioned one aspect (health or ecological) (21/46), with 3 participants identifying four dimensions, and 4 participants identifying 5 dimensions. The proportional relevance of the ecological dimension increased with number of dimensions mentioned. Other perceptions (not part of given definition) included; no connected to environment (n=7), i**ncorrect understanding of climate change (n=24) -** superficial with erroneous misunderstanding (+ others related to other dimensions such as low carb (n=8), low fat (n=21), high protein (n=8), low calorie (n=4), at risk of deficiency due to plant-based food (n=6), sustainability incompatible with the economy. | Health was the most prominent dimension of sustainability that was mentioned, however the ecological aspect was also commonly mentioned. There appeared to be a theme of self-centredness in understanding sustainability. Greater appreciation of wider perspective resulted in greater consideration of the ecological aspects Increase education about the potential for reduced environmental impact through consumer choice, with examples of effective interventions, support systems perspective. | Those that were included in the study were more likely to have an interest in nutrition/sustainability due to the recruitment method. |
| Gelinder, Hjälmeskog and Lidar, 2020^8^ | Sweden High school in a small town in Sweden, few parents have tertiary education, low academic achieving school (<60% of year nine students pass all subjects). The lessons included teacher descriptions, prompting and focus on sustainability (health, economic and environmental) in relation to food and cooking | Year 9 classes (n=2 classes; each for 2 different lesson) | Value Barriers/enablers | The study aimed to investigate students' decision making processing during a home economics classes and discuss barriers and enablers for teaching sustainable food consumption in schools. | Dewey's notions of learning, experiences and habits | Recruitment methods of the classes are unclear. Data was collected via video recording of lessons, regarding discussion of decision making between students. The lesson involved student preparing a 'sustainable' burger, with limited access to meat. Data was also collected from written protocols by students, justifying their ingredient choices in terms of sustainability (health, economy and environment). Data analysis included identification of decision making situation, transcription of discussions, and analysed in a deductive approach (using Practical Epistemological Analysis). | There were 16 incidences of food choices, only 3 related to sustainability (11 related to taste). Of the three regarding sustainability, 2 related to health (use wholemeal flour, include seeds), 1 related to cost (exclude onions). C**onsideration of environmental sustainability was made, however was not a determining factor in behaviour of food choices. Barriers to sustainable consumption (and education) include taste as a predominant factor, as well as past habits and experiences of normalised meat consumption. The complexity of sustainability, without adequate education, was also a barrier to understanding and making decisions based on sustainability.** | Either understanding of sustainability or the task was unclear, or taste was an overwhelming higher valued factor in food related decisions. | The understanding and value of sustainable foods was limited to the example of a burger (particularly choice and use of protein). |
| Geng, Liu and Zhu, 2017^9^ | China Eastern areas of China (high socioeconomic areas - Dalian, Shenyang, Shandgian and Sozhou) | Junior and senior high school students (n=623; n=284 from high schools, n=339 from junior high schools) | Value Barriers/enablers | The study aimed to examine adolescents' level of understanding of sustainable consumption, and how understanding may impact the motivation to understanding sustainable consumption behaviour. | Theory of planned behaviour | Participants were recruited, and questionnaires distributed and collected through the schools. Data was collected from the questionnaires pertaining to understanding of the concept of sustainable consumption, preferences and value of sustainable products, social norms, education sources regarding sustainability, and environmental awareness and attitude. This referred to the purchasing, use, and treatment and waste of products (not only food). Statistical analysis was undertaken to determine factors which impact upon behaviours of sustainable consumption. | Adolescents **prefer to purchase** sustainable products, but their consideration for the use and waste of sustainable products is reduced. Adolescents indicated that **knowledge of environmental benefits (in terms of effectiveness)** of specific products (eg. eco or organic labelling) may influence their purchasing choice.  **Awareness of and attitudes towards environmental sustainability, and education were also likely to influence choice.** | Education may have a motivational effect on sustainable consumption, however knowledge or labelling displaying the sustainability of products, and positive attitudes towards environmental sustainability may be more effective in the Chinese adolescent population to support sustainable consumption behaviours. | The purpose of this study was to assess sustainable consumption in a broad sense (not food only) which influenced the specificity of data collected. The values of Chinese adolescents may be different to those from other backgrounds. |
| Gisslevik, Wernersson and Larsson, 2019^10^ | Sweden A medium-sized school in middle-class region of Sweden. The school was selected as it had access to an equipped kitchen, supportive teacher and appropriate class sizes (<16 pupils). | Students aged 14-15 years (n=27) | Understanding | The study aimed to observe how students participate in and respond to education of sustainable development in home economics classes, and factors influencing this. | Ideal types (Convinced, Easy-going, Unable, Sceptical); with consideration of the 'width' and 'depth' of understanding. | The class was recruited purposively through a local network of home economics teachers.  Qualitative observational data was collected during the classes through field notes, audio (microphone attached to students) and video recordings, as well as through written assignments from students. Data was analysed thematically, with ideal types drawn from themes to describe and summarise student's perspectives and experiences. | **Almost all students displayed understanding of more tangible aspects (such as health impacts, organic production, cost etc),** however **few students displayed understanding of more complex, systematic aspects (eg. Global food systems, complexity of environmental impact).** Those who are **well informed, prepared and engaged were more likely to have greater depth and width in their understanding of sustainable food** (convinced). Those that were withdrawn, uninformed, unprepared with limited participation, were less likely to display depth or width of their understanding of sustainable food, or participate in discussions/questions. Student were more engaged when focussed on the tastiness of the produced meal rather than sustainability. | For long term sustainable consumers, education should support both values and the discussion of the justification for these values towards sustainability. This also is supported in that adolescents had a narrow perspective of sustainability, most closely related to themselves as individuals rather than broader community or global perspectives. | This study was part of an educational intervention, therefore other students in the context not exposed to the education, are likely to have a lower understanding of sustainable diets. |
| Greer, Davis, Sandolo, Gaudet and Castrogivanni, 2018^11^ | USA Bridgeport, Connecticut, is a large city with low median household income and high racial diversity. There is evidence for high levels of food insecurity in this area. | High school students (n=53) from 3 different local high schools. | Understanding Value Barriers/enablers | The study aimed to examine how high school students perceive local produce (fruit and vegetables). | Social Cognitive Theory | Participants were purposively sampled and invited to participate in the study. Focus groups were facilitated by researchers with a semi-structured guide, for approximately 60mins and were audio recorded. The guide focussed on topics of understanding and perceived value of locally grown foods, and barriers and enablers to purchasing locally grown food. Transcripts of the focus groups were analysed in duplicate for coding, with all researchers sampling a portion of the transcript and individually coding to check for consistency. Themes were derived from codes. | Local food was defined as **produced within the same state**, and student were able to identify some locally grown produce. Students were **commonly surprised when shown a list of locally grown products as they were unaware of what was/wasn't grown locally**. Students **valued the taste and freshness of locally grown produce**, and **perceived them as being better for the environment, community and economy**. Participants mentioned **cost** as a barrier to buying local produce, particularly when it was perceived to be organic, however some perceived local produce to be **lower in cost**. Some students suggested tasty local food to be available, and eye catching posters to promote local produce at school to increase consumption. | Taste is a primary motivator of food for students, however awareness and availability to local produce may increase consumption of local produce. Some students were motivated by the knowledge of reduced environmental and community impact and of local produce. | Minimal consideration of other aspects across the food system (eg. packaging, processing, waste etc). The study aims focus on local produce only (rather than sustainable diets). |
| Hallez, Vansteenbeeck, Boen and Smits, 2023^12^ | Belgium  Adolescents were recruited from various secondary schools in Flanders. | Young consumer aged 12-25 years (n=211, median age = 21 years; 73% female) | Value  Barriers/enablers | The study aimed to investigate the persuasiveness of visual and information packaging cues on the perceptions and choices of young consumers, in the context of sustainable food products. | Nil | Adolescents were recruited through the schools.  Participants were shown (online) four different snack products (ie chocolate bar, crips), of either a cool or warm colour. Data collected included participants’ product choice, willingness to eat or buy the product and perceived product sustainability, in addition to demographic and personal characteristics (including attitudes towards and value of sustainable food choices). Data was analysed statistically. | **Cooler colours and an ecological claim in product packaging was perceived as more sustainable** (and healthy). However packaging colours and ecological claims did not influence willingness to purchase or consume snack. Older participants were less likely to perceive snacks as healthy or sustainability. Younger participants reported significantly **less guilt about snacks with an ecological label**. | Cooler colours (green/blue) and ecological claims are associated with more sustainable (and healthier) products. However these perceptions did not influence willingness to purchase or consume the snacks, potentially due to mistrust of labelling. | Age of ‘younger participants’ is not defined. Convenience sampling and high female participation rate may not reflect broader population. |
| Havermans, Rutten and Bartelet, 2021^13^ | Netherlands Two schools in the Province of Limburg, which borders Belgium and Germany. This area had a population of ~1.2million people. Students aged 16 often have a job outside of school | Students aged 15-16 years (n= 11; 7 girls) | Understanding Value Barriers/enablers | The study aimed to examine how adolescents intend to (or not) adopt a plant-based diet | Reasoned Action Approach | Purposive sampling was used to recruit participants from secondary schools. Participants were asked to bring 2-6 pictures of food to individual, semi-structured interviews. Each interview lasted between 18-30minutes, during which a guide was used by interviewers to gather information from participants regarding their attitudes, perceived social norms and perceived behavioural control regarding based on the adoption of a plant-based diet. Photos were used to prompt discussions and interviews were audio-recorded. Transcripts of interviews were analysed with a deductive approach, using codes derived from the theoretical framework. | Most participants (10/11) **reported low or no awareness of the benefits of a plant based diet**, with **some uncertainty as to its definition**. **Some benefits noted related to the environment** and health. **Most (7/11) had little or no intention to adopt a plant-based diet.** Some concerns noted included **taste and appearance** of plant-based foods and limited motivation. Most students had high self-efficacy regarding the adoption of a plant-based diet. Students reported a strong parental beliefs about their diets, but denied strong consideration of their family or friends preferences when making personal dietary choices. Eating meat was the **perceived social norm**. Most participants regularly ate fruit, vegetables and grains. All participants indicated eating meat every day with only 2 attempting to limit this. | Future interventions should go beyond increasing knowledge, but towards facilitating behaviour change through the shift of intentions. Taste of foods for adolescents is most important for acceptance and exposure may assist with this in future food systems interventions | Participants were not blinded to the specific topic of the study, therefore introduces some bias in the sample. Participants were unable to bring photos to discuss in the interview. Some lack of depth regarding the components of a plant-based diet and their perceptions of this (eg. pulses, meat alternatives etc), and also perception of overall sustainable diets (appropriate for the study aim). |
| Kazmierczak-Piwko, Kulyk, Dybikowska, Dubicki and Binek, 2022^14^ | Poland  Students attending primary schools. | Children and adolescents (9-15 years old) (n=1326) | Barriers/enablers | The study presents results from primary data regarding Polish adolescent young consumers’ awareness and functioning patterns of consumption, and recommendations for the development of sustainable consumption patterns amongst children and adolescents | Nil | Recruitment details were not reported.  Participants completed a survey with two parts; firstly a questionnaire with a focus on the ability to choose environmentally sustainability products, and pro-environmental, domestic behaviours (including shopping and eating).  Statistical analysis was undertaken to compare results between age groups. | Factors influencing food product choice include product composition (52%), price (65%), product feedback (20%), environmentally friendly packaging (20%), brand (20%), country of origin (17%), popularity (13%), product markings/symbols (12%), attractive packaging (6%) and advertising (3%). | Increase awareness of environmental impact of products and how to identify sustainable products. Encourage parents to involve children and adolescents in product choice. Promotion of eco-labelling. | Data regarding factors influencing adolescent’s food choice was not reported separately from children. It is unclear whether this relates to sustainability, or food choices in general. |
| Lim, Lim, Leong and Le, 2021^15^ | Malaysia No further details were provided | Late adolescents (aged 15-19yo) (n=25 in the interviews, n=5 in the focus group) | Barriers/enablers | The study aimed to examine how and why adolescents waste food | Taxonomy model of disposition decisions | The participants were recruited using non-probability sampling, from public places. Data was collected through 1) individual interviews exploring how and why food is wasted (approximately 45mins each), and 2) focus group discussions to validate findings from the interviews (approximately 1 hour). Data was analysed thematically. | Five themes are discussed regarding how food is wasted: 'keep it for original purpose', 'leave it for other people to dispose of', 'convert it for another purpose' (eg. pet food, different meal), 'try and finish it', 'get rid of it permanently'. Reasons for these include food related factors (**cost, taste, health of the food**), socio-cultural (**etiquette, social norms**), and personal factors (**value of the environment, finances, hunger, low awareness of environmental impact of waste, low perceived responsibility for responsible waste**) | Increase awareness of the value of food waste on the environment, and responsible consumption, particularly in institutions such as schools. | The study context and description of participants was vague. Potentially due to similar cultural beliefs (eg. strong family/community values, a larger sample size would not reach saturation as quickly. |
| Lindgren, 2020^16^ | Sweden School in a large urban municipality, high socioeconomic area in a private school | Secondary school students aged 16-18 years (n=67; 29 females) | Value | The study aimed to explore students' meaning of meat consumption formed, and these views are formed with access to animal products is disrupted. | Dislocatory intervention | The sampling method of participants is not reported. Data was collected through focus groups (25-45 mins each) and one interview. An interview guide was used to encourage discussion about eating sustainable foods, and opinion to the school initiative to impose a vegan month. Transcripts were analysed from the discussions. | Opinions were influenced by **political opinion** (left vs right) of the students and their political perceptions of the school (viewing veganism as leftist), gendered politics (viewing veganism as feminine, targeted for girls), embodied reaction (eg. posters, surveys, newspaper articles etc), and conflict of personal rights (taking away freedom of choice). | Complete removal of animal-based products induced political conflict. Communication and avoiding a top-down approach with interventions may alleviate some conflict. | Specifically focussed on avoiding animal based foods only. Specific population (high achiever's in upper secondary school in Sweden, mostly females who identify as vegetarian/vegan) |
| Miller, Barwood, Furfaro, Boston, Smith and Masek, 2021^17^ | Australia High school students across different socioeconomic indices, at the completion of the 'Nutrition Transformation Games' | High school students in grades 7-10 (n=206) from 5 different schools. | Value | The study aimed to identify food and nutrition related topics of interest to Australian adolescents | Not specified | Participants were selected using non-random sampling technique. Data was collected using a questionnaire as students selected which topics (of 16) were of interest for future nutrition-education. | Topics with the highest interest include chronic disease prevention, maintaining healthy weight, the right foods for active lifestyles, food safety and how to prepare healthy foods. Topics relating to food insecurity, work hunger and **food waste (particularly in higher socio-economic contexts and for boys) were also of interest**. Topics of less interest were natural foods, vitamin supplements, **vegetarian foods, choosing local foods**, influences of food choice and advertising. | Students are interest in food waste and food security, however are more interested in healthy food. Education should focus on healthy eating, with consideration of food waste and food insecurity. | It is unclear what education the students have already received and were wanting further education for themselves or for the future curriculum. |
| Ojala, 2022^18^ | Sweden Medium sized central Swedish municipalities, specifially adolescents that previously scored highly with a climate-friendly choice questionnaire. The participants were well educated (backgrounds in natural science, social science and arts), mostly with Swedish backgrounds. | Older adolescents aged 17-19 years (n=15) | Barriers/enablers | The study aimed to explore conflicts and coping strategies towards adolescents' climate friendly food choices | Transformative learning | Participants were sampled from the results of a previous questionnaire, in a strategic and convenience manner, of those that reported climate-friendly eating habits. Data was collected using individual interviews via Zoom, using an interview guide. Data was analysed thematically. | Climate friendly food choices were driven by **previous education (for example at school), family habits, complementing values (health, animal rights), social norms.** Difficulties include **social conflicts** (eg. with family values especially if living with parents), cultural/religious conflict, **taste** (preference of meat, dairy or imported produce), c**onvenience, hunger, economic cost** (especially when buying local products), **product availability, lack of knowledge, doubt of efficacy, and healthy compromises** (eg. deficiency, mental health). Coping strategies include **independence with food preparation, sharing personal beliefs, maintain broad perspective of impact, moral and identity arguments, make realistic goals.** | Future education can include potential conflicts/barriers that may arise for young people to discuss, particularly in the structured, formal format. | Small sample of older adolescents that are already making climate-friendly choices. |
| Plummer, Wilson, Yaneva-Toraman, McKenzie, Mitchell, Northover, Crowley, Edwards and Richards, 2022^19^ | Caribbean Diverse setting with participants from the UK and Caribbean (English-speaking areas) | Youth aged 14-20 years (n=25) | Understanding Value Barriers/enablers | The study aimed to examine the role of Afrodescendant and Indigenous culinary and agricultural heritage in climate action youths and perceptions of food security. | Not specified | Participants were recruited through the Caribbean Youth Environment Network and Black Open University (UK). Data was collected as from participants in each session using focus groups discussions regarding their diet, climate-change awareness, and the role of traditional foods and climate resilience. Data was analysed thematically. | **Participants were concerned about climate change on the local agricultural environment.** Commonly **price** was reported as a barrier to consuming local foods. **Motivators for local and sustainable food practices include health/healing, caring for nature, and communal/collectivist cultural norms**. Participants shared knowledge and beliefs about the value of **local and sustainably grown food** (including traditional foods and food practices, communal preparation and consumption of foods, natural/less processed foods) through an emotive song. | The value of sustainable foods of young Caribbean Indigenous people was strong, particularly with motivators of community and caring for nature. | Shared focus across the agricultural and consumer roles regarding sustainable food. |
| Ronto, Ball, Pendergast and Harris, 2016^20^ | Australia Schools offering home economics as a subject at high school; 2 private schools and 1 public school. | Adolescents in high school (aged 12-17 years; 69% female) (n=131) | Understanding Value | The study aimed to explore adolescents' perspectives of the importance of food literacy, and which aspects are valued | Not specified | Participating schools were recruited through home economics teachers, via email. All students in each high school were invited to participate. Data was collected using focus groups between 30-60 minutes in duration. Facilitators presented a list of 22 aspects of food literacy, and asked students to rank 6 most (then least)important aspects of food literacy for health. Students responses were shared and collated. Data was analysed quantitatively and qualitatively through coding of transcripts from discussions. | The most commonly rated important aspects of food literacy include food safety and hygiene, un/healthy foods, appropriate portion sizes, dietary guidelines and animal welfare, and having a positive attitude towards cooking and healthy eating. **Environmental sustainability was rated higher by males and students in the middle years (than senior years**). Least important aspects were related to shopping and cooking skills. Students required clarification for the terms **'environmental sustainability'** and 'animal welfare**'.** Once explained, these terms were related to what was used to **feed animals, being a vegetarian, and food miles or locally grown produce**. There was a reported disconnect between food knowledge and putting them into practice. | There is some confusion about the relationship between the environment and food systems, and the term 'environmental sustainability', in relation to food literacy for Australian adolescents | The results from this study capture a range of ages, but from a single school. Environmental sustainability may not have been explained/defined in all groups. |
| Serra-Mallol, Wacalie, Nedjar-Guerre, Wattelez, Frayon and Galy, 2021^21^ | France New Caledonia is diverse culturally, environmentally, economically and in language with a high prevalence of obesity. | Adolescents in middle schools aged 11-16 years (n=29 in interviews; 375 in group discussions) | Understanding Value Barriers/enablers | The study aimed to explore how families with 11-16 year old children define 'eating well'. | Not specified | The recruitment method is not reported. Data was collected from individual interviews (45-60 mins) and group discussions with adolescents. Questions in the interview focussed on food consumption, and perceptions of 'eating well', whereas group discussions focussed on perceptions only. Data was transcribed and analysed thematically. | Adolescents reported 'eating well' to be related to nutrition (in the context of health), personal taste, **local provenance,** quantity and commensality. Regarding local provenance, there was a **focus on locally grown products (including organic) and traditional foods, and positive perception of these.** Barriers to familial/local production, although perceived as organic and natural, include **geography and social responsibilities.** | Include aspects of local foods in public health messaging, as this is valued by the local community. | Perceptions of 'sustainable diets' may be different to 'eating well'. |
| Uhlmann, Ross, Buckley and Lin, 2022^22^ | Australia  Brisbane high schools with varying nature/urbanisation and food-related activities but similar socioeconomic and cultural diversity. | High school students aged 11-18 years (n=59 completed surveys from >3000 students invited; n=32 completed implicit association test) | Understanding  Value | The study aimed to identify relationships between adolescent nature relatedness and wellbeing, and explore adolescents’ connection toward nature and food | Not specified | Participants were recruited through high schools.  Data was collected through an online survey (20min – includes demographic information, family cohesion, food growing exposure, food concern, diet quality, healthy eating attitudes, personal wellbeing and nature relatedness) and ‘implicit association test’ (explain decision making processes and demonstrate understanding – audio and visual data was collected regarding participants explanations of terms and relationships).  Survey data was analysed statistically. IAT data was analysed by frequency and thematic analysis. | Rural students reported a closer association to nature than students from the city, and also a closer relationship between all three concepts (food, me, nature). Overall, most participants (>90%) had a closer relationship to the concept food than nature, however there was acknowledgement that food comes from nature. **Although rural and agricultural school students more commonly linked food with nature, and reported spending time in/enjoying nature, city students uniquely raised concerns about food production on nature.** | Building self-efficacy may support healthy food choices and therefore promote food concern and consideration of the environmental when making food related decisions. Education may be able to shape attitudes and behaviours through providing motivation and opportunities. | Very low participations rates suggests biased results and limited sample scope. |

**References**

S1. Bailey C, Prichard I, Drummond C, Drummond M. Australian adolescents’ beliefs and perceptions towards healthy eating from a symbolic and moral perspective: A qualitative study. *Appetite*. 2022;171105913. doi:10.1016/j.appet.2022.105913

S2. Bersamin A, Izumi BT, Nu J, O'Brien D M, Paschall M. Strengthening adolescents' connection to their traditional food system improves diet quality in remote alaska native communities: Results from the neqa elicarvigmun pilot study. *Translational Behavioural Medicine*. 2019;9(5):952-961. doi:10.1093/tbm/ibz087

S3. Collins A, Galli A, Patrizi N, Pulselli FM. Learning and teaching sustainability: The contribution of ecological footprint calculators. *Journal of Cleaner Production*. 2018;174:1000-1010. doi:10.1016/j.jclepro.2017.11.024

S4. Colombo PE, Elinder LS, Patterson E, Parlesak A, Lindroos AK, Andermo S. Barriers and facilitators to successful implementation of sustainable school meals: A qualitative study of the optimat (tm)-intervention. *International Journal of Behavioral Nutrition and Physical Activity*. 2021;18(1)89. doi:10.1186/s12966-021-01158-z

S5. Damen FWM, Steenbekkers B. Added value of physical food products as a stimulus during interviewing. *Appetite*. 2022;169:105819. doi:10.1016/j.appet.2021.105819

S6. Derler H, Berner S, Grach D, Posch A, Seebacher U. Project-based learning in a transinstitutional research setting: Case study on the development of sustainable food products. *Sustainability*. 2020;12(1)233. doi:10.3390/su12010233

S7. Dornhoff M, Hornschemeyer A, Fiebelkorn F. Students' conceptions of sustainable nutrition. *Sustainability*. 2020;12(13)5242. doi:10.3390/su12135242

S8. Gelinder L, Hjälmeskog K, Lidar M. Sustainable food choices? A study of students’ actions in a home and consumer studies classroom. *Environmental Education Research*. 2020;26(1):81-94. doi:10.1080/13504622.2019.1698714

S9. Geng DY, Liu JJ, Zhu QH. Motivating sustainable consumption among chinese adolescents: An empirical examination. *Journal of Cleaner Production*. 2017;141:315-322. doi:10.1016/j.jclepro.2016.09.113

S10. Gisslevik E, Wernersson I, Larsson C. Pupils' participation in and response to sustainable food education in swedish home and consumer studies: A case-study. *Scandinavian Journal of Educational Research*. 2019;63(4):585-604. doi:10.1080/00313831.2017.1415965

S11. Greer AE, Davis S, Sandolo C, Gaudet N, Castrogivanni B. Formative research to create a farm-to-school program for high school students in a lower income, diverse, urban community. *Journal of School Health*. 2018;88(6):453-461. doi:10.1111/josh.12627

S12. Hallez L, Vansteenbeeck H, Boen F, Smits T. Persuasive packaging? The impact of packaging color and claims on young consumers’ perceptions of product healthiness, sustainability and tastiness. *Appetite*. 2023;182:106433. doi:10.1016/j.appet.2022.106433

S13. Havermans RC, Rutten G, Bartelet D. Adolescent's willingness to adopt a more plant-based diet: A theory-based interview study. *Front Nutr*. 2021;8:688131. doi:10.3389/fnut.2021.688131

S14. Kazmierczak-Piwko L, Kulyk P, Dybikowska A, Dubicki P, Binek Z. Sustainable consumption among children and adolescents. *Production Engineering Archives*. 2022;28(3):257-267. doi:10.30657/pea.2022.28.32

S15. Lim TY, Lim B, Leong CM, Le AD. Finish your plate! Food disposition behaviour among late adolescents. *British Food Journal*. 2021;123(9):3192-3207. doi:10.1108/BFJ-03-2021-0329

S16. Lindgren N. The political dimension of consuming animal products in education: An analysis of upper-secondary student responses when school lunch turns green and vegan. *Environmental Education Research*. 2020;26(5):684-700. doi:10.1080/13504622.2020.1752626

S17. Miller M, Barwood D, Furfaro M, Boston J, Smith S, Masek M. Identifying differences in nutrition-related learning interests of adolescent students. *Journal of School Health*. 2021;91(4):277-284. doi:10.1111/josh.12997

S18. Ojala M. Prefiguring sustainable futures? Young people's strategies to deal with conflicts about climate-friendly food choices and implications for transformative learning. *Environmental Education Research*. 2022;28(8):1157-1174. doi:10.1080/13504622.2022.2036326

S19. Plummer N, Wilson M, Yaneva-Toraman I, et al. Recipes for resilience: Engaging caribbean youth in climate action and food heritage through stories and song. *Sustainability*. 2022;14(14)8717. doi:10.3390/su14148717

S20. Ronto R, Ball L, Pendergast D, Harris N. Adolescents' perspectives on food literacy and its impact on their dietary behaviours. *Appetite*. 2016;107:549-557. doi:10.1016/j.appet.2016.09.006

S21. Serra-Mallol C, Wacalie F, Nedjar-Guerre A, Wattelez G, Frayon S, Galy O. 'Eating well' in pacific islands countries and territories: A qualitative and normative approach to food cultures in new caledonia. *Appetite*. 2021;163:105192. doi:10.1016/j.appet.2021.105192

S22. Uhlmann K, Ross H, Buckley L, Lin BB. Nature relatedness, connections to food and wellbeing in australian adolescents. *Journal of Environmental Psychology*. 2022;84:101888. doi:doi.org/10.1016/j.jenvp.2022.101888
