# Supplementary material for: Toward Sustainable Diets—Interventions and Perceptions Among Adolescents: A Scoping Review
Source: Nutr Rev. 2024 May 29;83(2):e694–710. doi: 10.1093/nutrit/nuae052 (PMC11723159; doi:10.1093/nutrit/nuae052)
Supplement: nuae052_Supplementary_Data [file nuae052_supplementary_data.zip › nuae052_Supplementary_Data/Supplementary information C - Summary table interventions V2.docx]

## Supplementary information - Appendix C: Data extraction table – intervention studies to improve the environmental sustainability of adolescents’ diets

| Citation | Context (country and setting) | Participants (age, number) | Study aims and intervention components | Intervention type | Sustainable diet principles* | Theory or framework | Study design and methods | Key findings | Conclusions/Recommendations | Limitations |
| --- | --- | --- | --- | --- | --- | --- | --- | --- | --- | --- |
| Bersamin, Izumi, Nu, O'Brien D and Paschall, 2019^1^ | Alaska Remote, native Alaskan communities (low income, off road, predominantly local/traditional foods, distributed independently, at risk of food insecurity – schools procure shelf-stable/processed foods via plane to supplement locally grown produce) | Middle and high school students, average age 14yo (n=76) | The study aimed to evaluate a food systems intervention on diet quality, intake and attitudes and beliefs. The study involved a school-based intervention across three domains; 1) cafeteria – increase access to local salmon (weekly), 2) classroom – 5 X culturally responsive lessons (benefits of traditional diet, increase ability to choose healthy and environmentally sustainable foods that correspond with community values), and 3) 4 X community events celebrating traditional foods (Intergenerational Iron Chef Contest). The control group received the classroom component after the 4-month data collection for community equality and all schools received the same menu (due to a centralised food system structure) | Classroom teaching Cooking Food procurement (local) Parent/community engagement Presentation of learning | 4, 9, 10, 14, 15 | A framework was developed with the local community, based on Social Cognitive Theory which identified 9 main themes (including behavioural capability, collective efficacy, outcome expectations and self-efficacy). | Pre-post study with control.  All students at the local middle and high schools were invited to participate. Self-reported data was collected pre- and post- intervention (baseline, at 4 months and at 9 months after commencement, across one academic year). Data collected included fish intake (measured using a 24hr recall and blood biomarker), diet quality (Healthy Eating Index) and enculturation. A survey was conducted following the study to explore attitudes and beliefs regarding health and wellness (perceptions of salmon benefits, impact of eating traditional foods, attitude towards environmental impact of food, attitudes towards and and value of having required food skills). | There was an **increase in local fish consumption** in intervention groups (0.3 to 1.3 serves in past 24hr, increase in biomarker from 8.2% to 8.6%). Differences in attitudes and perceptions were insignificant at baseline (intervention vs comparison) but increased with time during the intervention (control group decreased fish intake over the same time). | Making changes in the food supply chain in conjunction with education can facilitate increased local food consumption, both in attitudes and behaviour. | Students in the control group had significantly less total fish intake at baseline, and reported a higher level of enculturation (“white way of life”). |
| Black, Velazquez, Ahmadi, Chapman, Carten, Edward, Shulhan, Stephens and Rojas, 2015^2^ | Vancouver  Public elementary and high schools in Vancouver, some (61%) of which participated in Think&EatGreen@School (program includes aspects of sustainable food including production, consumption, preparation and procurement, learning about the whole food cycle, research and developing policy and programs) | Elementary and high schools (Total n=33; high schools n=15). Median high school size was 1294 students per high school. | The study aimed to develop the School Food Environment Assessment Tool, use it to assess schools in Vancouver, and identify opportunities to improve food environments. In this way, there was an assessment of interventions/ programs already in place. | Classroom teaching Food gardens Composting Food availability (local, organic, seasonal, plant-based foods) Reduce waste/packaging | 2, 3, 4, 9, 10, 11, 12, 13, 15 | Comprehensive School Health Framework (macro, and physical/social environments, teaching and learning, partnerships/services) | Cross sectional study.  Purposive sampling was used to recruit schools for geographical and socioeconomic diversity Data was collected 1) from physical food environment (availability, eating spaces, presentation, food available), 2) through interviews with teachers/food staff (current policies, programs, procurement, sales), 3) through interviews with vice/principals (initiatives, broader goals). The School Food Environment Assessment Tool was developed. Domains of the tool include gardens, composting, food preparation activities, sustainable food related teaching/learning, and availability of healthy and sustainable foods). Assessment of healthy food was determined using Canada’s Food Guide, and food sustainability was determined by availability of foods that are minimally processed, locally grown/processed, seasonal, organic, consider vegetarian options, decreased packaging waste) | Median score for availability of healthy food was 1/5 (range 1-3); sustainable food was 1/5 (0-2), gardens 1/5 (0-3), composting 2/5 (0-3), food preparation activities 2/5 (1-2), teaching/learning activities 1/5 (1-3).  Regarding sustainability, initiatives include using **local, organic, or school garden produce, minimally processed/packaged food, and including vegetarian options**. Most schools made an effort to offer sustainable foods (28/33), however only two schools offered sustainable options regularly (**none were regular/always**) | Develop clearer definitions of ‘sustainable food’ options and resources and education for those involved in food services (eg. for procurement and menu design). Ongoing use and refinement of the SFEAT tool. | No recorded consideration of which school/s had been involved in certain initiatives or programs. |
| Cabot, Bosch, Barcelo-Oliver, Cabello, Torrens-Serra, Miro and Delgado, 2019^3^ | Spain Mostly attendees from the local area were young couples with young children. | Attendees were mostly adolescents (n=580; 50% of total). Activity participants and those involved in data collection (n=200 adolescents; 40% of total attendants) | The study aimed for undergraduate students to use experimental learning experiences to connect conventionally taught concepts in the classroom to real world problems, and engage with the community to share their learnings and promote recycling of kitchen leftovers. Adolescents were involved as the tertiary students shared their findings from their activities including a worm composter, measuring soil contents and toxic elements, produced posters and 5min presentations, at a public fair. | Public teaching | 10, 13 | Not specified | Case study.  Participants were recruited voluntarily as attendees at the fair as audience members were invited to take part in a quiz. Data was collected from participants following presentations from the quiz to measure understanding, as well as a survey to evaluate the effectiveness of the presentations to drive behaviour change. Further details of data recruitment, data collection and analysis are not explained. | Adolescents were more difficult to engage in the presentations than younger children or adults. **Adolescents were more distracted or cynical of the presentations and scored lower in the quiz** (27% of adolescents vs 8% of primary school aged children scored poorly). | Public, short presentations were more effective with young children and adults than adolescents, for engagement and understanding regarding food waste recycling. | Limited detail was provided in the paper regarding study methods, background of participants and, therefore, limiting the application of findings. |
| Collins, Galli, Patrizi and Pulselli, 2018^4^ | UK and Italy Cardiff University (UK), School of Geography and Planning, specifically students in a postgraduate unit relating to research measures of environmental impact. University of Siena (Italy) offered a sustainability course for all students and employees, and high school apprentices. The course focusses on environmental consequences of consumption. In this study, the high school students were in their third or fifth year at a local Scientific High School (general education with scientific focus), or third year at a Technical High School (specialising in laboratory teaching) | Post graduate (n=20) and high school (n=31; 74% male) students | The study aimed to assess the ecological footprint of students, and its calculation as an educational tool. The intervention involved a ~2hr group session with participants, in which they received education about using the ecological footprint calculator, completed the calculation for themselves (across different categories such as food, mobility, goods, services etc), reflected on the results and categories with the highest footprint, discussed strategies to reduce their footprint and re-calculated the footprint. Finally there was a discussion about the effectiveness of the calculator as an educational tool. | Classroom teaching | 9, 10 | Not specified | Case study. Participants were recruited through the university and the intervention was included as part of their studies. Data collected included the ecological footprint (as global hectares derived from National Footprint Accounts, Consumption Land-Use Matrix (eg. carbon, grazing land, cropland etc) and National Footprint Accounts by consumption category at a geographical level) for each student as a reflection of their current lifestyle and with consideration of potential lifestyle changes.  Results were compared between age groups, across categories, and before and after theoretical lifestyle changes. | Most high school students had a higher ecological footprint than post-grad students, and the national average.  Carbon accounted for the largest proportion of the footprint (61-63% of footprint), followed by cropland use then forest land. Food was the largest category contributing to ecological footprint (average 40%).  Following re-calculation, a reduction of 15-19% of the footprint was obtained by high school students – largely with changes to ‘mobility’ and ‘food’. High schoolers were surprised about the large contribution from ‘food’, and perceived this as difficult to change (suggested that a change was needed in the supply chain, not consumer choice). **Only half of students were willing to change their diet to reduce the ecological footprint.** Suggested changes included decrease meat consumption, decrease packaging, swap to organic and/or locally produced foods. There was reluctance from participants to change to strict vegetarian or vegan diets. The calculator was user-friendly, informative and engaging, however lacked some sensitivity for specific products or activities. | Calculators and group discussion assist with students’ understanding of the environmental impact of their lifestyle in a personalised manner. Using appropriate language and user-friendly tools enable this to be effective, however it is unclear what age groups would find this most beneficial. Future studies should focus on how similar interventions result in behaviour change (rather than theoretical ideas). | No clear methodology for data collected regarding the effectiveness of using the footprint calculator as an educational tool. No report on age of high-school apprentices, however likely older, well-educated adolescents. The calculator used data from Switzerland, therefore the environmental impact scores would be less accurate in either Italy or UK contexts. |
| Colombo, Elinder, Patterson, Parlesak, Lindroos and Andermo, 2021^5^ | Sweden Stockholm schools (Grade 0-9), 52% of local population were not Swedish, high proportion of parents were without tertiary education. | Students in grade 5 and 8 (n= 29), and kitchen staff (n=13) | The study aimed to explore student and staff’s experiences, and barriers and enablers to the implementation of and optimised school lunch menu. The intervention (OPTIMAT study) involved the design of an optimised menu (optimised for nutrient content, greenhouse gas emissions, cost and similarity to the previous menu), without any adjunct promotion or education for students. | Food availability | 2, 3, 4, 9, 13, 14, 15 | Not specified | Case study.  All kitchen staff from the three schools were invited to participate (n=14). Recruitment of students was through selection of names from class lists.  Focus groups were conducted for a duration of 52-75mins for kitchen staff and 24-50mins for students.  Topics included; general perceptions of school lunch, experiences receiving menu, perception of sustainable diet, barriers/enablers of increased plant based meals.  Analysis was completed with an inductive approach with transcriptions from the focus groups. Each transcript was read multiple times, and codes, subcategory and category groupings were developed by the research team | Staff found the new menu challenging to adopt **(time, cost, taste, management of leftovers, resources and practice for new dishes, communication/engagement with stakeholders, perceived negative perceptions of students**). Time for planning helped overcome this. Mixed response to the taste of the new menu, **common negative reports from pupils**. Some favourite meals include pasta dishes, Mexican tortillas, vegetarian burgers, lentil dishes (mixed easily with meat dishes). **Greater acceptance without ‘vegetarian’ labels (diet identity/ taste perceptions), and familiar foods (eg. lasagne), highlight benefits, gradual change**. | Include students and kitchen staff in menu design to increase acceptance. Need for supportive environment (increase knowledge, resources, inspiration, stakeholder engagement). | One municipality only, in Sweden. |
| Derler, Berner, Grach, Posch and Seebacher, 2020^6^ | Austria High school students attending a polytechnical school, or nutrition and agriculture school | Secondary school students aged 15-19 years (n=117) | The study aimed to enhance high school students competencies related to food, food technology and sustainability.  The intervention involved students taking part in research methods workshops (including focus groups discussions, assessment of dietary habits with food diaries and images, menu design for personas), design and optimisation of sustainable food product ideas (including experimental nutrition and sensory analysis), and presentation of results. Researchers and teachers were available to support students throughout the study to optimise their products. | Classroom teaching Cooking Presentation of learning | 6, 10, 11, 14 | Problem-based learning: knowledge, understanding, applying | Case study.  The method of recruitment is not discussed. Data collected includes dietary intake (using a pictorial and written food diary, and survey), and product specification sheets from the developed sustainable products (not discussed as a result). Data collection methods from focus groups was not disclosed. The dietary intake from food diaries was compared to the national dietary guidelines. | Ten percent of students met daily fruit and vegetable recommendations and nearly two thirds ate fast foods at least weekly. Students reports homemade, regional and seasonal food were important to them. Five final products were developed: energy bites, cheese chips, smoothie, cereal bars and pasta muffins – details of the products were not reported however ingredients were suggested to be **locally sourced.** Students were challenged and learnt to balance competing aspects of sustainability food (eg. cost, nutrition, environmental impact etc) | Problem-based learning can be used to engage students in the development of products in an interactive manner. | Data reporting the effectiveness of the program (eg. Sustainability of products created, qualitative data from students, level of knowledge or understanding) is limited from the study. |
| Eustachio Colombo, Elinder, Lindroos and Parlesak, 2021^7^ | Sweden Three Stockholm schools (Grade 0-9), 52% of local population were not Swedish, high (51%) proportion of parents were without tertiary education. | All students were recipients of the intervention (n=1635 students) Participants in data collection regarding satisfaction were in years 5 and 8 only | The study aimed to implement and evaluate an optimised four-week menu. The intervention (OPTIMAT study) involved the design of an optimised menu (optimised for nutrient content, greenhouse gas emissions, cost and similarity to the previous menu), without any adjunct promotion or education for students. | Food availability | 2, 3, 4, 6, 9, 13, 14, 15 | Social Cognitive Theory | Pro-post study design without control.  School were recruited through meeting of public meal managers, and then invited school chefs and schools to express interest. Data was collected through plate waste audits (at baseline for 4 weeks, and during the 4 week intervention; using the Swedish Food Agency’s method to measure waste, and the School Food Sweden’s method for measuring consumption – weighed records of kitchen waste, prepared waste, serving waste, leftover waste and plate waste). Meal satisfaction data was collected from students using the online School Food Sweden tool (regarding overall satisfaction, frequency of school lunch consumption, satiety, and wastage). Qualitative data was analysed using appropriate software. | There was no significant difference in plate, serving or consumption waste between baseline and the intervention in any of the schools. The implemented menu reduced GHGE by 40%, cost 14% less and met most nutritional (97% energy and iron, 99% selenium and 98% of zinc) requirements, whilst maintaining a similar food profile to the baseline diet. This menu had 6 (of 40) vegetarian dishes (increased from 4) (total increase in vegetables by 7%, increase fast/oils 5%, decrease seafood 13%, decrease fruit/berries 54%, decrease meat 32%, decrease seasonings 26%, decrease dairy 13%, decrease cereal 5%).  Waste fluctuated day to day, likely reflecting taste preferences of the menu items. There were no statistically significant difference in satisfaction between baseline and the intervention. However this was a low level of satisfaction. | Including students and staff in designing acceptable menus may decrease plate waste and increase satisfaction. However with a single strategy (food availability), the environmental impact of school lunches can be reduced. | The ages of students is not clear, however young adolescents participated in the intervention and were key participants in data collection. The sustainability consideration were relatively narrow (GHGE only) and based upon data that may be inaccurate. |
| Figueroa-Pina, Chavez-Servin, de la Torre-Carbot, Caamano-Perez, Lucas-Deecke, Roitman-Genoud and Ojeda-Navarro, 2021^8^ | Mexico Quetero high school (private school) | Students from grade 6-12 (n=126; n=42 in each intervention and the control group. A sub-group of the food gardens and education group also participated in structured interviews (n=10) | The study aimed to evaluate the effectiveness of education, in conjunction with gardens, on improving fruit and vegetable intake.  The intervention involved students participation in 19 X 60 minute education lessons in the classroom (topics included sustainable agriculture, food security, food research, animal farm, school food audit), with an additional 10 X 60minute lessons in the garden (some topics include garden set up, compost, seeds, pest prevention and control, and harvesting and cooking) for those in the garden and education group. A control group did not take part in any of these classroom or garden lessons. | Food gardens  Classroom teaching Presentation of learning Parent engagement | 2, 3, 4, 9, 10, 11, 13, 14, 15 | Cognitive constructivism | Randomised control trial.  Participants were recruited from one school, and randomised into one of two intervention groups, or the control group. Data was collected from participants using a 3-day food diary and fruit and vegetable food frequency questionnaire before and after the intervention. These were used to evaluate fruit and vegetable, as well as macronutrient intake, of participants. The Pro Children qualitative questionnaire was used to evaluate psychosocial factors (personal cognitive skills, attitudes, preferences, perceptions of environment, perception of barriers and their social environment). Structured interviews were undertaken with students participating in the gardens assessing their opinions, knowledge, feelings, social environment and willingness to apply knowledge from the gardens at home. Appropriate statistical analysis was undertaken. | Students involved in both the food gardens and education **increased their average consumption of fruit and vegetables** (+65g/d greater than control group) and frequency of consumption (4.1d/wk; +1.4d/wk from baseline), more-so than those that received the education only (+45g/d greater than control group; 4days/wk, +1.2d/wk from baseline) Students participating in the garden and classroom education reported an **increased awareness of environmental impact associated with the food system**. Students participating in the gardens **enjoyed the program and were willing to (or already had) start their own gardens at home**. | Education in the classroom and garden (29 lessons) were more effective at increasing awareness of sustainable food production and consumption than education (19 lessons) alone with Mexican adolescents. | Qualitative data regarding knowledge and attitude changes was not collected from the classroom education group. Differences in dietary behaviour may be a reflection of the increased exposure to education, rather than exposure to the garden. |
| Fulford and Thompson, 2013^9^ | Canada Winnipeg (low-income neighbourhoods, high chronic disease, low education, discrimination, food insecure) | Youth, aged 9-18 years (n=7), and program staff (n=3) | The study aims were not specified. The intervention through the Youth EcoAction Program involved farm visits, seed starting, transplanting, gardening, food prep, workshops, gardens. The program also included broader youth mentoring, collaboration between schools, volunteers and several community organisations. | Public Teaching Food gardens Cooking Farm trips Community engagement Presentation of learning Composting | 2, 3, 9, 11, 13, 14 | Circle of Courage (belonging, mastery, independence, generosity) | Case study.  All interns of the program (n=8) were recruited for participation in semi structured interviews. Details of data collection methods and analysis are not clearly described in the manuscript however data appears to be analysed deductively in relation to the Circle of Courage model. | Increased **knowledge of the food system** (including regarding organic and local foods, use of chemicals in production, and global food production).  Increase in food security (individually and also community wide, sharing of resources and knowledge with other local youths).  Most youths reported an **increase in fruit and vegetable intake**. Increase in environmental awareness and behaviour (decreased littering (both individual and community), **increased composting**, recycling, decreased chemical use with food production, increased reported value of the environment)  Increase in skills, self-esteem and community building. | Within the staffing and funding limits, the program was effective in support behaviour change relating to food waste and consumption of healthy foods, as well as knowledge regarding the environmental impact of food systems. | Limited information regarding data collection and analysis. |
| Gisslevik, Wernersson and Larsson, 2019^10^ | Sweden A medium-sized school in middle-class region of Sweden. The school was selected as it had access to an equipped kitchen, supportive teacher and appropriate class sizes (<16 pupils). | Students aged 14-15 years (n=27) | The study aimed to observe how students participate in and respond to education of sustainable development in home economics classes, and factors influencing this. The intervention involved 14 X 180minute lessons with topics including food resource management and waste, reducing meat, portion sizes, and using local and organic products. | Classroom teaching Cooking | 2, 4, 6, 7, 8, 9, 13 | Ideal types (Convinced, Easy-going, Unable, Sceptical); with consideration of the ‘width’ and ‘depth’ of understanding. | Case study.  The class was recruited purposively through a local network of home economics teachers.  Qualitative observational data was collected during the classes through field notes, audio (microphone attached to students) and video recordings, as well as through written assignments from students. Data was analysed thematically, with ideal types drawn from themes to describe and summarise student’s perspectives and experiences. | Students had greater understanding of more tangible aspects (such as health impacts, organic production, cost etc) than more complex, systematic aspects (eg. Global food systems, complexity of environmental impact). Those who are well informed, prepared and engaged were more likely to have greater depth and width in their understanding of sustainable food (convinced). Those that were withdrawn, uninformed, unprepared with limited participation, were less likely to display depth or width of their understanding of sustainable food, or participate in discussions/questions. Student were more engaged when focussed on the **tastiness** of the produced meal rather than sustainability. | For long term sustainable consumers, education should support both values and the discussion of the justification for these values towards sustainability. There should be a respectful place for discussions to deepen the understanding and attitudes towards sustainability. Hands-on education with food enables students to readily translate and apply their skills, given the available time and resources. This also is supported in that adolescents had a narrow perspective of sustainability, most closely related to themselves as individuals rather than broader community or global perspectives. | The effectiveness of the intervention on behaviour change was not reported. |
| Jones, Dailami, Weitkamp, Kimberlee, Salmon and Orme, 2012^11^ | UK The program was implemented across >3600 schools (primary, secondary and special) in England. This study specifically targeted schools that received extra funding for the program, across a range of socioeconomic contexts. | Secondary schools (n=24) | The study aimed to understand barriers and enablers of the implementation of ‘Food for Life Partnership’ programme. This program involved components relating to school food policies, food procurement, meals/catering design, food gardens, cooking skills, and farm links. | Classroom teaching Food gardens Cooking Food procurement Policy Farm trips Parent/community engagement | 2, 3, 9, 10, 11, 12, 13, 14, 15 | Food for Life Partnership programme strands | Pre-post study design without control.  Purposive sampling was used to select at least 3 schools from each of the regions taking part in the program. Data was collected from school staff with evidence of program related outputs. Data was collected from students at baseline and 18-24-month follow-up regarding attitudes toward food and sustainability issues with a piloted survey. Data was collected from school staff through semi-structed questionnaires at baseline and follow up regarding perceptions, barriers and enablers to the program’s implementation. Data was analysed in relation to the programme strands and indicators. | Increase in all schools across food leadership (policy), quality and provenance (availability), education, and parent and community engagement. **No significant Increase in fruit and vegetable consumption, however increase in involvement in growing fruit and vegetables and ability to prepare meals.** **No significant change in attitudes or consideration towards sustainable foods**. **Positive attitude towards sustainable food and food behaviours was significantly associated with increase fruit and vegetable consumption.** Staff perceived the program to be highly effective with the main barriers reported relating to **restricted time and costs, and paperwork**. The most successful aspects were shift in whole school food culture, promotion of healthy eating and cooking education. The least successful were networking with catering organisations and staff, the community, other schools and parents. | Recommendations for future interventions to consist of overarching policies and goals, in conjunction with specific and discrete, project-driven interventions that can be evaluated. | Wide sample population with varying resources, background etc, therefore the program was delivered differently to different schools. Depth and characteristics of each schools intervention were unclear |
| Kowalewska and Kollajtis-Dolowy, 2018^12^ | Poland Cities in Poland (Warsaw, Ciechanow, Ostrowia Mazowiecka) | Students from 11 schools aged 14 years (n=555) | The study aimed to identify the effects of education on the reduction of food waste. The intervention involved a group of participants watching a 3-4 minute educational video on food waste and its prevention. The same group, in addition to a second group were provided with a food waste related leaflet for their parents. Finally a control group (with no educational intervention) was included. | Classroom teaching Parent engagement | 2, 3, 4, 6, 13 | Not specified | Pre-post study design with control.  Schools were randomly selected to participate in the study. Data was collected using a three-day food record (also accounting for wasted food) including types of foods and fluids with estimated portion sizes. A questionnaire was also used to assess knowledge of food waste (at baseline, after watching the video education (for one sub-group) and 3 months after baseline. | Leaflet to parents increase students’ knowledge regarding menu planning and prevention of waste. Students **who received both the leaflet and video education had ~twice the effect on increasing knowledge both short term (with all aspects) and long-term (regarding quantities of food waste, menu planning, and prevention of food waste)** | Targeting both children and parents resulted in better increase in knowledge and behaviour related to food waste. |  |
| Lombardini and Lankoski, 2013^13^ | Finland Schools in Helsinki were approved to offer a vegetarian only day at school by the local council with much local interest and opposition due to the removal of freedom of choice relating to food. Previously, a vegetarian option was previously always available for students. It is optional for students to received lunch at school. | Schools (primary and upper-and lower-secondary) (n=33) with additional control group of schools (n=10) | The study aimed to assess the impact of food choice restriction (vegetarian only options) on plate waste in schools. The intervention involved 33 schools (centralised food system for all schools) provided vegetarian only food for students one day each week. | Food availability (vegetarian) | 2, 3, 4, 6, 13, 15 | Not specified | Pre-post study design without control.  Schools were recruited through the food procurement company (mandatory for some schools). Data was collected using pre- and post- intervention waste measures, each time over 5 days. Number of students receiving school lunches, amount of food taken, and amount of food wasted was measured at baseline, after 11 exposures to the vegetarian only days, and after 23 exposures to the vegetarian only days. Data was analysed using statistical software. | In the fist 11 weeks, in upper-secondary schools, waste increased by 89% and in lower-secondary schools, participation decreased by 19%, food taken decreased by 11% and plate waste increased by 40%. After 23 weeks, in upper secondary schools, there was a 26% decrease in amount of food taken, and 16% decrease in lower-secondary students participation. However, **there was no change in plate waste, and consumption of vegetarian meals in lower-secondary schools increased.** | Particularly for lower-secondary students, longer term exposure appears to increase consumption of vegetarian options. | The study was implemented in 2011, during which time the food system may have been different to current times in terms of available products, perceptions/attitudes of consumers, distribution methods etc. |
| Necca, Tamino and Santovito, 2014^14^ | Italy One junior-middle school each in Teolo (rural, with most student’s having access to fresh fruit and vegetables from home gardens or local farms) and Selvazzano | Four classes of students (n=86; n=22 in the control group, n=21 in the Active Learning group, n=23 + 20 in the Assessment of Learning intervention group) | The study aimed to assess the effectiveness of using ‘Assessment for Learning’ as a methodology for teaching secondary school students about sustainable food. A control group taught students using a didactic, lecture mode, where as the Assessment for Learning group involved discussions, brain-storming and structed feedback during the class from the teacher. The active learning techniques involve discussions, brainstorming and group activities (without structured teacher feedback). | Classroom teaching | 2, 3, 4, 7, 9, 10, 12 | Not specified | Case study.  Purposive sampling was used to select schools for participation. Quantitative data was collected from teachers regarding different teaching styles, and grades of assessment were used to compare results between classes. | On average, students achieved higher grades when using Assessment for Learning methods (average grade 8.95) or Active Learning (average grade 8.33), than lectures (average grade 8.22). | Engaging students in discussions and feedback may facilitate better learning on the topic of sustainable foods. | It is unclear if there are statistically significant differences in the results from different teaching styles. The sample size from each class is small, and may introduce bias through the sampling of different schools. It Is unclear how these grades may translate into behaviour of the adolescents. |
| Plummer, Wilson, Yaneva-Toraman, McKenzie, Mitchell, Northover, Crowley, Edwards and Richards, 2022^15^ | Caribbean Diverse setting with participants from the UK and Caribbean (English-speaking areas) | Youth aged 14-20 years (n=25) | The study aimed to examine the role of Afrodescendant and Indigenous culinary and agricultural heritage in climate action youths and perceptions of food security. The Recipes for Resilience project involved storytelling (with maps), online games, talks and music to discuss climate change, and ultimately write a song. | Teaching Creative arts Presentation of learning | 2, 9, 10, 14, 15 | Not specified | Case study.  Participants were recruited through the Caribbean Youth Environment Network and Black Open University (UK). Data was collected as from participants in each session using focus groups discussions regarding their diet, climate-change awareness, and the role of traditional foods and climate resilience. Data was analysed thematically. | Participants were concerned about climate change on the local agricultural environment. Commonly price was reported as a barrier to consuming local foods. **Participants shared knowledge and beliefs about the value of local and sustainably grown food through an emotive song.** The song was then able to be performed at a global conference. | Using culturally appropriate learning and communication methods can contribute to bridging the gap between knowledge and action | This method of teaching may be better suited to some cultures than others. Shared focus across the agricultural and consumer roles regarding sustainable food. |
| Prescott, Burg, Metcalfe, Lipka, Herritt and Cunningham-Sabo, 2019^16^ | USA Two Colorado schools with nutrition programs | 6^th^ grade students (age =11-12) (n=268) | The study aimed to examine the impact of student-driven education and promotion on adolescents food selection, consumption and waste (esp of fruit and vegetables) at school, as well as the impact on students’ knowledge and attitude towards the food system. The intervention involved lessons incorporated into the curriculum (food system, environmental impact of food, food waste, food processing and school cafeteria waste), leading to poster creation by students to share with older students about their learnings. | Food environment Classroom teaching Presentation of learning Creative arts | 2, 3, 9, 10, 12, 13, 14 | Self-determination theory (autonomy, competence and relatedness needs to impact motivation quality) | Pre-post study design. Schools were recruited through science educator networks via email. Data was collected from students regarding their plate waste (quantified estimates from photos, monthly for sixth months), knowledge and attitudes towards foods systems via a survey (pre- and post- classroom lessons), photographs of created posters, and regarding most popular posters (student voting). Interviews with teachers were also undertaken for feedback of the intervention. Interviews and poster content was coded thematically. | Barriers to implementation included time constraints for teachers Facilitators of the intervention included flexibility with the curriculum, support and training for teachers. Themes from the students work include food waster prevention and prevention of packing waste/use of disposable food items. **Significant increase in knowledge/attitudes towards food waste. No other reported significant change in knowledge or attitudes.** Intervention students wasted less fruit and vegetables (especially for students with access to salad bar) | Education through the curriculum and student-driven promotion may reduce food waste and increase awareness of the environmental impact of food systems. Overall, with support, the teachers found the intervention feasible. | Some confounding factors – decreased food waste may be related to ability to choose own portion size |

*Sustainable dietary principles according to FAO&WHO (2019) in summary: 1 – support breastfeeding, 2-consume minimally processed foods, 3-consume whole, plant-based foods, 4-consume moderate amounts of animal products, 5-consume clean water, 6-adequate energy consumption, 7-reduce diet-related diseases, 8-food safety, 9-maintain environmental impact within targets, 10-preseve biodiversity, 11-avoid antibiotics and hormone use in production, 12-minimze plastics, 13-reduce food waste, 14-culturally appropriate food systems, 15-food accessibility and desirability, 16-gender equity^17^

**References**

S1. Bersamin A, Izumi BT, Nu J, O'Brien D M, Paschall M. Strengthening adolescents' connection to their traditional food system improves diet quality in remote alaska native communities: Results from the neqa elicarvigmun pilot study. *Translational Behavioural Medicine*. 2019;9(5):952-961. doi:10.1093/tbm/ibz087

S2. Black JL, Velazquez CE, Ahmadi N, et al. Sustainability and public health nutrition at school: Assessing the integration of healthy and environmentally sustainable food initiatives in vancouver schools. *Public Health Nutrition*. 2015;18(13):2379-91. doi:10.1017/s1368980015000531

S3. Cabot C, Bosch R, Barcelo-Oliver M, et al. Experimental learning experiences orchestrated by undergraduate college students to actively engage middle school studnets in food waste recycling. 2019:6637-6643.

S4. Collins A, Galli A, Patrizi N, Pulselli FM. Learning and teaching sustainability: The contribution of ecological footprint calculators. *Journal of Cleaner Production*. 2018;174:1000-1010. doi:10.1016/j.jclepro.2017.11.024

S5. Colombo PE, Elinder LS, Patterson E, Parlesak A, Lindroos AK, Andermo S. Barriers and facilitators to successful implementation of sustainable school meals: A qualitative study of the optimat (tm)-intervention. *International Journal of Behavioral Nutrition and Physical Activity*. 2021;18(1)89. doi:10.1186/s12966-021-01158-z

S6. Derler H, Berner S, Grach D, Posch A, Seebacher U. Project-based learning in a transinstitutional research setting: Case study on the development of sustainable food products. *Sustainability*. 2020;12(1)233. doi:10.3390/su12010233

S7. Eustachio Colombo P, Elinder LS, Lindroos AK, Parlesak A. Designing nutritionally adequate and climate-friendly diets for omnivorous, pescatarian, vegetarian and vegan adolescents in sweden using linear optimization. *Nutrients*. 2021;13(8)doi:10.3390/nu13082507

S8. Figueroa-Pina DG, Chavez-Servin JL, de la Torre-Carbot K, et al. Evaluation of the effect of a school garden as an educational didactic tool in vegetable and fruit consumption in teenagers. *Nutrition Research and Practice*. 2021;15(2):235-247. doi:10.4162/nrp.2021.15.2.235

S9. Fulford S, Thompson S. Youth community gardening programming as community development: The youth for ecoaction program in winnipeg, canada. *Canadian Journal of Nonprofit and Social Economy Research*. 2013;4(2):56-75.

S10. Gisslevik E, Wernersson I, Larsson C. Pupils' participation in and response to sustainable food education in swedish home and consumer studies: A case-study. *Scandinavian Journal of Educational Research*. 2019;63(4):585-604. doi:10.1080/00313831.2017.1415965

S11. Jones M, Dailami N, Weitkamp E, Kimberlee R, Salmon D, Orme J. Engaging secondary school students in food-related citizenship: Achievements and challenges of a multi-component programme. *Education Sciences*. 2012;2(2):77-90. doi:10.3390/educsci2020077

S12. Kowalewska MT, Kollajtis-Dolowy A. Food, nutrient, and energy waste among school students. *British Food Journal*. 2018;120(8):1807-1831. doi:10.1108/bfj-11-2017-0611

S13. Lombardini C, Lankoski L. Forced choice restriction in promoting sustainable food consumption: Intended and unintended effects of the mandatory vegetarian day in helsinki schools. *Journal of Consumer Policy*. 2013;36(2):159-178. doi:10.1007/s10603-013-9221-5

S14. Necca IZ, Tamino G, Santovito G. Sustainable food: An educational proposal for key stage 3 in secondary schools based on the assessment for learning methods. 2014:7348-7356.

S15. Plummer N, Wilson M, Yaneva-Toraman I, et al. Recipes for resilience: Engaging caribbean youth in climate action and food heritage through stories and song. *Sustainability*. 2022;14(14)8717. doi:10.3390/su14148717

S16. Prescott MP, Burg X, Metcalfe JJ, Lipka AE, Herritt C, Cunningham-Sabo L. Healthy planet, healthy youth: A food systems education and promotion intervention to improve adolescent diet quality and reduce food waste. *Nutrients*. 2019;11(8)doi:10.3390/nu11081869

S17. Food and Agriculture Organization, World Health Organisation. *Sustainable healthy diets - guiding principles*. 2019. <https://www.fao.org/3/ca6640en/ca6640en.pdf>
