# Supplementary figures and images for: Toward Sustainable Diets—Interventions and Perceptions Among Adolescents: A Scoping Review
Source: Nutr Rev. 2024 May 29;83(2):e694–710. doi: 10.1093/nutrit/nuae052 (PMC11723159; doi:10.1093/nutrit/nuae052)

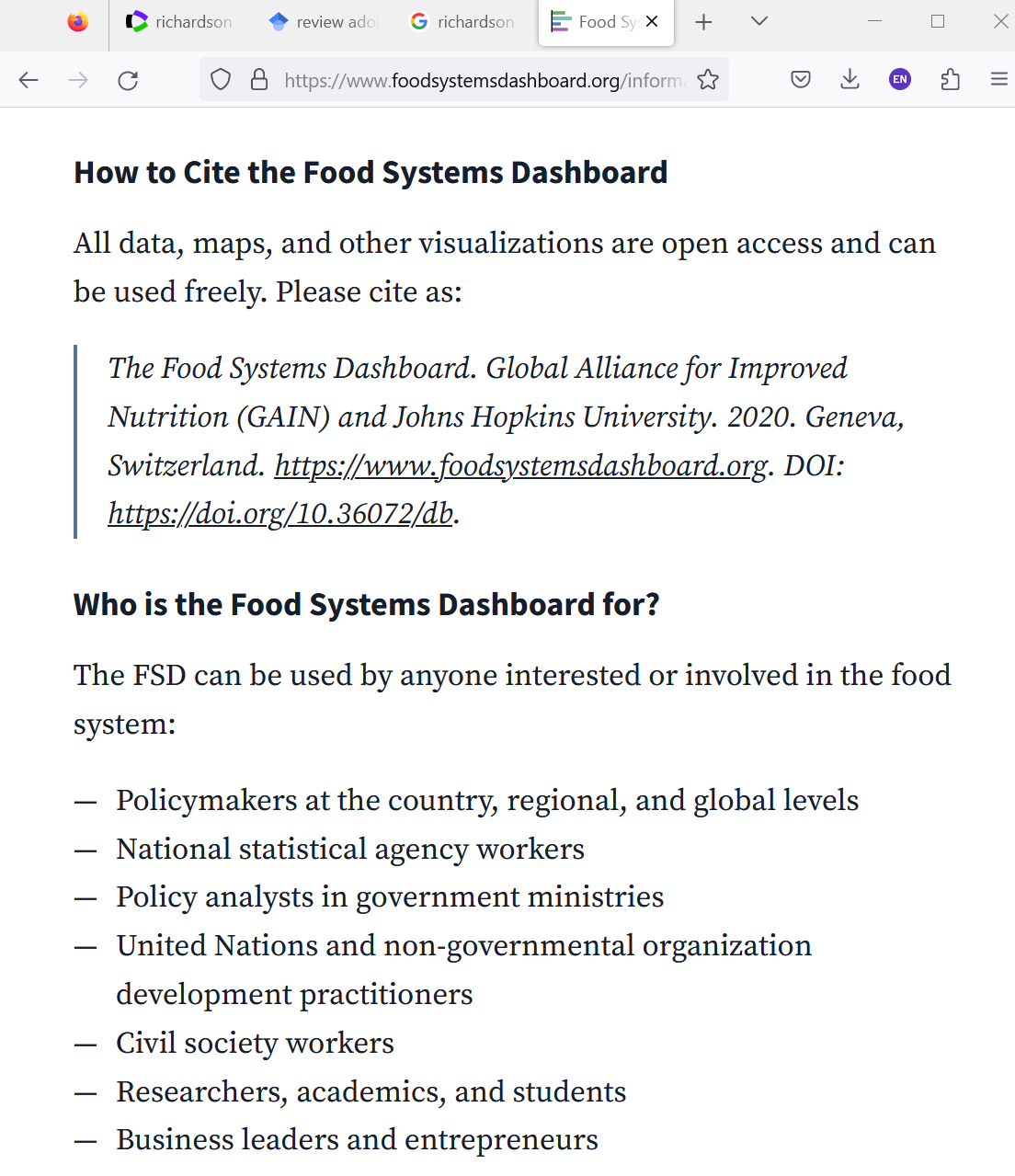

Supplement: nuae052_Supplementary_Data [file nuae052_supplementary_data.zip › nuae052_Supplementary_Data/Evidence of approval of Food Systems Dashboard for use.png]
